# Site-specific *N*-glycosylation of integrin α2 mediates collagen-dependent cell survival

**DOI:** 10.1016/j.isci.2021.103168

**Published:** 2021-09-25

**Authors:** Yen-Lin Huang, Ching-Yeu Liang, Vera Labitzky, Danilo Ritz, Tiago Oliveira, Cécile Cumin, Manuela Estermann, Tobias Lange, Arun V. Everest-Dass, Francis Jacob

**Affiliations:** 1 Ovarian Cancer Research, Department of Biomedicine, University Hospital Basel and University of Basel, Hebelstrasse 20, Basel 4031, Switzerland; 2 Institute of Anatomy and Experimental Morphology, University Cancer Center Hamburg, University Medical Center Hamburg-Eppendorf, Hamburg20246, Germany; 3 Proteomics Core Facility, Biozentrum, University of Basel, Basel4056, Switzerland; 4 Institute for Glycomics, Griffith University, Gold Coast Campus, Gold Coast, QLD4222, Australia; 5 Adolphe Merkle Institute, University of Fribourg, Fribourg1700, Switzerland

**Keywords:** Cell biology, Proteomics, Cancer

## Abstract

Integrin alpha 2 (ITGA2) promotes cancer metastasis through selective adhesion to ECM proteins; however, the specific contribution of integrin glycosylation remains uncertain. We provide evidence that ITGA2 is a highly glycosylated transmembrane protein expressed in ovarian cancer tissue and cell lines. In-depth glycoproteomics identified predominant *N*- and *O*-glycosylation sites harboring substantially divergent ITGA2 glycosylation profiles. Generated putative ITGA2 *N*-glycosite mutants halted collagen and laminin binding and cells lacking *N*-glycosylated ITGA2 were marginally adherent to collagen, likely associated with its enhanced proteasome degradation through poly-ubiquitination. Proteomic and enrichment pathway analysis revealed increased cellular apoptosis and collagen organization in non-glycosylated ITGA2 mutant cells. Moreover, we provide evidence that ITGA2-specific sialylation is involved in selective cell-ECM binding. These results highlight the importance of glycans in regulating ITGA2 stability and ligand binding capacity which in turn modulates downstream focal adhesion and promotes cell survival in a collagen environment.

## Highlights

• In-depth glycoproteomics reveal divergent ITGA2 glycosylation
• Site-specific N-glycans regulate protein stability and ECM ligand binding affinity
• Loss of N-glycosylation induces proteasome degradation through poly-ubiquitination
• N-glycosylation mediates collagen-dependent cell survival through focal adhesion

## Graphical abstract

**Figure:**
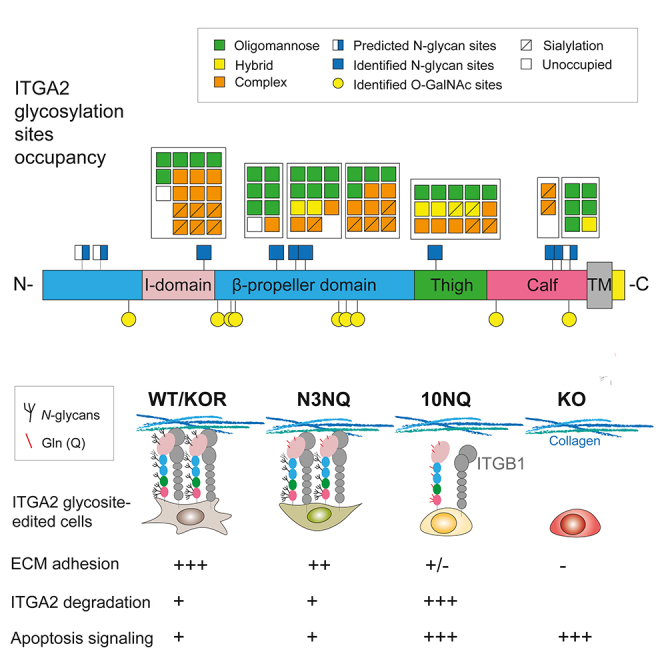

## Introduction

Protein glycosylation is one of the most common post-translational modifications found in all domains of life. Glycosylation plays crucial biological roles in protein folding and quality control, protein trafficking, and secretion as well as ligand recognition (Moremen et al., 2012). Glycans are mainly attached to the polypeptide chain through amide linkages to asparagine (Asn) or glycosidic linkages to serine/threonine referring to *N*- and *O*-glycosylation, respectively. The initial attachment of oligosaccharides to the Asn within the consensus NXT sequence (-Asn-X-Ser/Thr-) takes place in the endoplasmic reticulum (Breitling and Aebi, 2013). The subsequent processing of the oligosaccharides continues in the Golgi apparatus giving rise to the three main types of *N*-glycans including oligomannose, hybrid, and complex types based on the composition of oligosaccharides and branching features. However, unlike *N*-glycosylation, our knowledge about the functional roles of O-glycosylation is still limited due to technical constraints primarily related to lack of enrichment strategies and predictive consensus sequence motifs (Vester-Christensen et al., 2013). As glycan structures are highly diverse and altered in response to various biological processes such as cancer-associated cell survival, adhesion, and migration, there is a need to understand how glycosylation is regulated through a repertoire of glycosyltransferases and glycan-modifying enzymes along the biosynthetic pathway (Narimatsu et al., 2019). Therefore, emerging studies investigate the correlation between glycan and gene expression profiles and multiple high-throughput approaches targeting specific glycosyltransferases in cancer (Costa et al., 2020). Recent advances in mass spectrometry-based analytical methodologies also offer comprehensive glycan profiling allowing to better understand the contribution of glycosylation to biological regulation in specific cellular transitions and disease states (Moremen et al., 2012). However, the function of site-specific glycosylation of a particular protein in a functional context remains one of the major challenges in the field of glycobiology.

Integrins are heterodimeric transmembrane receptors mediating signal transduction between cell-cell and cell surrounding environment factors such as the extracellular matrix (ECM). The combination of 18 α and 8 β subunits generates a total of 24 functional integrin heterodimers in humans which are involved in a wide range of biological functions such as immune response, cellular differentiation, survival, cell adhesion, and migration (Campbell and Humphries, 2011). Structural information revealed that integrins undergo a conformational rearrangement as a result of headpiece extension and leg domain separation to enhance ligand binding affinity (Springer and Dustin, 2012). Given the fact that both α and β integrin subunits carry high abundance of *N*-glycans, it is of great interest to understand how glycosylation may influence the conformational change and thus the activation or inactivation of integrins upon ligand recognition (Cai et al., 2017).

Early studies have shown that ligand-binding and cell-surface transportation of integrin is inhibited in the presence of the *N*-glycosylation inhibitor, tunicamycin (Chammas et al., 1991). In addition, treatment of purified integrins with peptide-N-glycosidase F (PNGaseF) the enzyme that cleaves these glycans, blocked the association of α5 and β1 subunits, leading to a concomitant loss of fibronectin-binding activity (Zheng et al., 1994). These results suggest that *N*-glycosylation is essential to the functional expression, cell spreading, migration, ligand binding, and heterodimer formation of integrin α5β1 and presumably other integrins. However, the detailed mechanisms by which *N*-glycosylation site-specifically mediates these functions remain unclear. Isaji et al. firstly demonstrated that three glycosylation sites on the I-like domain of β1 integrin represent the minimal *N*-glycosylation required for functional expression and the formation of heterodimer α5β1 (Isaji et al., 2009). Although the determination of *N*-glycan structures is very cumbersome and challenging, Nakagawa et al., have initially identified 23 sialylated and 10 neutral *N*-glycan structures on integrin α5β1 from the human placenta using three-dimensional HPLC mapping (Nakagawa et al., 1996). A predominant complex type of *N*-glycans on integrin α3β1 subunits was also identified by matrix-assisted laser desorption/ionization mass (MALDI-MS) in human ureter epithelial cells (Litynska et al., 2002). Although it is known that conformational rearrangement by glycosylation is recognized as an important mechanism for controlling the affinity of ligand binding by integrins (Luo et al., 2003), the role of individual *N*-glycosylation sites on integrins remains to be investigated.

Considering the fact that altered expression of integrins and aberrant glycosylation on the cell surface has been associated with tumorigenesis and metastasis in multiple cancers (Desgrosellier and Cheresh, 2010; Marsico et al., 2018), a comprehensive characterization of glycans on integrins in cancer would provide valuable insight into understanding the molecular basis of integrin-dependent signaling and ligand binding function in cancer. Therefore, in this study, we aim to dissect the functional role of *N*-glycan sites on integrin, with the focus on collagen-binding integrin alpha 2 (ITGA2), which has been recently shown to exhibit a multifaceted role in regulating cancer progression and metastasis in ovarian (Huang et al., 2020), melanoma (Baronas-Lowell et al., 2004), gastric (Matsuoka et al., 2000), colon (Bartolome et al., 2014), and breast cancers (Ramirez et al., 2011).

## Results

### ITGA2 is a highly abundant cell surface glycoprotein on ovarian cancer cells derived from tissue and ascites

**Figure 1:**
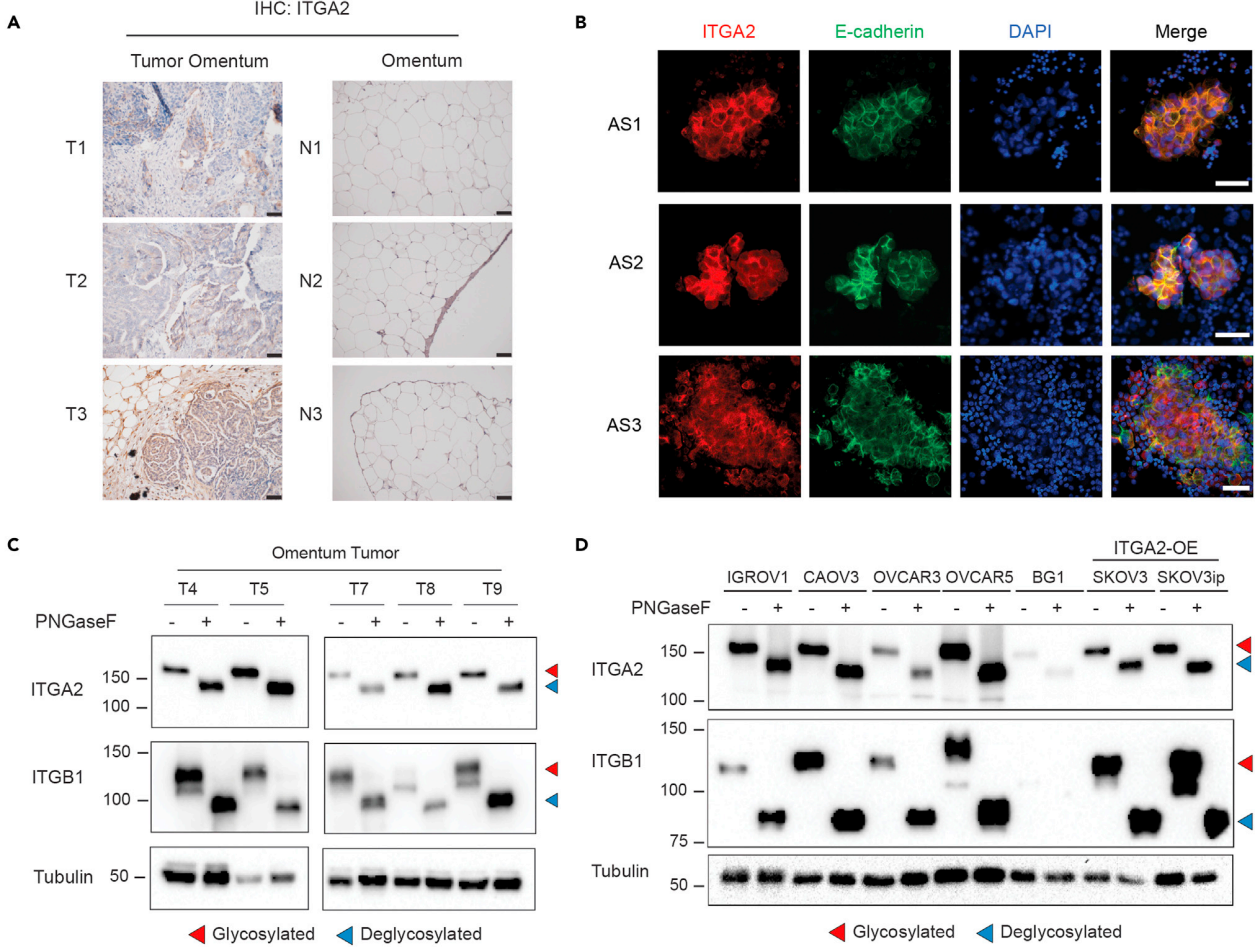
ITGA2 is highly expressed and *N*-glycosylated in ovarian tumor cells (A) Representative IHC staining of normal and metastatic omentum for ITGA2. Scale bar 50 μm. (B) Representative immunofluorescence images with membranous E-cadherin (green) and ITGA2 (red) staining in ascites-derived tumor spheroids from advanced ovarian cancer patients. Scale bar 50 μm. (C and D) Protein lysates of metastatic tumor omentum and (D) ovarian cancer cell lines (n = 7) treated with or without PNGaseF for Western blot analysis. Red arrow indicates glycosylated protein, blue arrow deglycosylated protein with a remarkable mobility shift in the Western blot.

We have previously suggested a new route of peritoneal dissemination through the interaction of integrin alpha 2 (encoded by *ITGA2*) with collagen I and III (Huang et al., 2020) usually enriched in the omental tumor of ovarian cancer patients (Pearce et al., 2018). Here, we show that ITGA2 is markedly upregulated in omental tumors compared to normal omentum by assessing the tissue expression (Figure 1A). In addition, the presence of epithelial ITGA2+ tumor cells in patient-derived ascites supports the hypothesis on its role in promoting peritoneal dissemination to the collagen-rich niche at the omentum (Figure 1B). Considering the critical role of glycosylation on multiple integrins in cancer (Marsico et al., 2018), we examined the presence of *N*-glycosylation of ITGA2 in ovarian tumor tissues (n = 6) and cancer cell lines (n = 7) by peptide-*N*-glycosidase F (PNGaseF) treatment. We identified a significant mobility shift (∼30kDa) in all of the investigated tumor samples and cancer cell lines (Figures 1C and 1D). These findings suggest that *N*-glycosylation is ubiquitously and abundantly present on ITGA2 in ovarian cancer cells.

### Capturing site-specific ITGA2 glycosylation with targeted glycoproteomics

**Figure 2:**
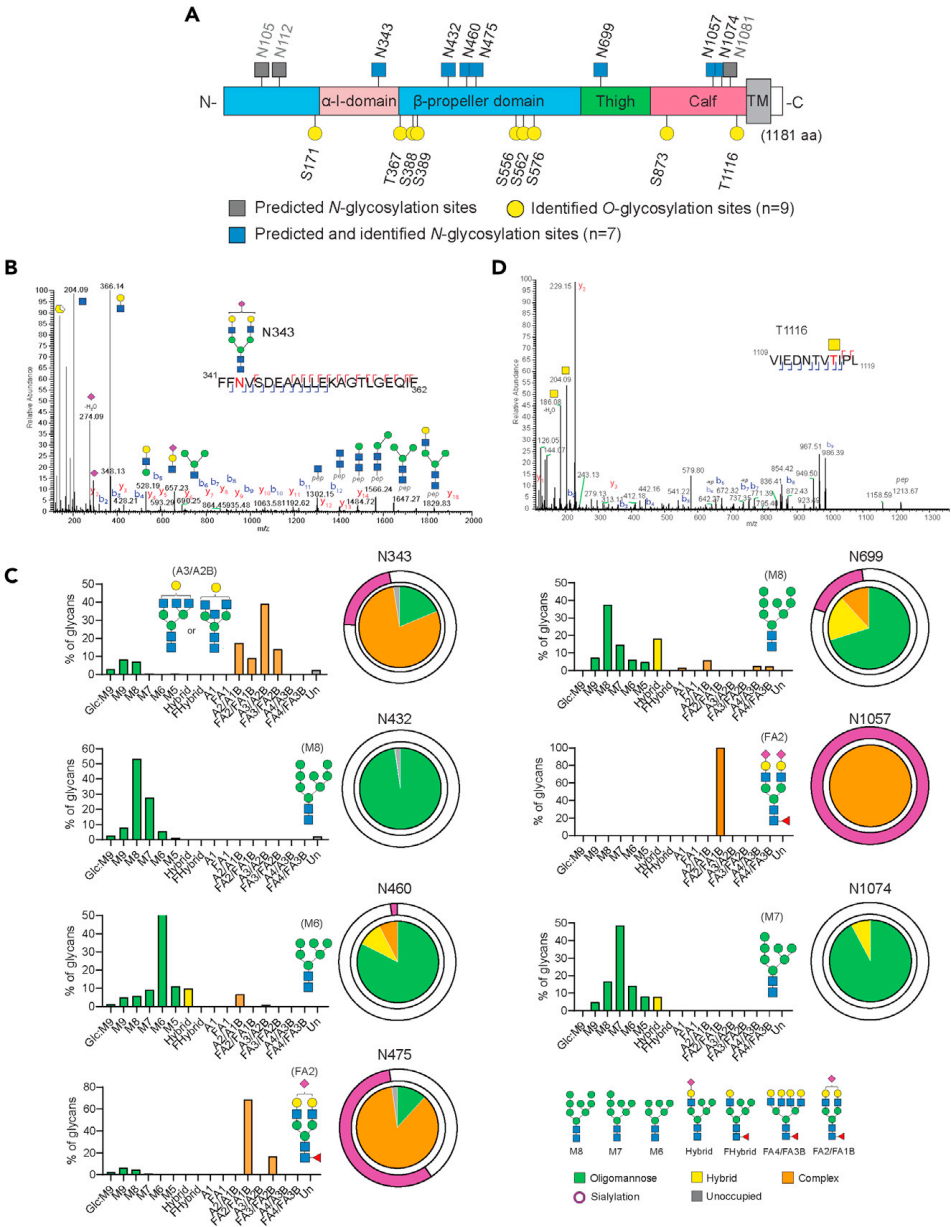
Characterization of site-specific ITGA2 N-glycosylation (A) The schematic illustrates structural domains of ITGA2 and the site occupancy of N-glycans. Color code indicates the predominant glycan types from oligomannose- and complex-type glycans. (B) LC-MS/MS-based identification of glycopeptides is shown as MS spectra corresponding to N-glycosylation site N343. The representative HCD MS2 spectrum is shown to exemplify its identification of glycan composition. (C) Graphs summarize quantitative MS spectrometric analysis of the glycan population present at individual *N*-glycosylation sites simplified into categories of glycans. Most abundant glycan in each site is annotated. Oligomannose-type glycan series (M9 to M5; Man9GlcNAc2 to Man5GlcNAc2) is colored green, hybrid-type glycans (hybrid and F hybrid) are yellow, and complex glycans are grouped according to the number of antennae and presence of core fucosylation (A1 to FA4) and are colored orange. Unoccupancy of an N-linked glycan site is colored gray. Pie charts summarize the quantification of these glycans. (D) LC-MS/MS-based identification of *O*-glycosylation site T1116.

Initial *in silico* prediction revealed 10 potential *N*-glycosylation sites at asparagine and 12 *O*-glycosylation sites at serine and threonine on human ITGA2 using NetNGlyc 1.0 Server and NetOGlyc 4.0 Server, respectively (Figure S1). To experimentally validate the *N*-glycosylation sites of ITGA2, the protein lysate of ovarian cancer cells expressing hemagglutinin (HA)-tagged ITGA2 was immunoprecipitated with anti-HA magnetic beads, resolved by SDS-PAGE and the corresponding ITGA2 region was excised and subjected to in-gel digestion with either trypsin or chymotrypsin (FiguresS2A and S2B). The mass spectrometric characterization of glycoproteins poses several significant analytical challenges primarily due to the microheterogeneity of the glycoforms and reduced ionization efficiencies (Yu et al., 2018). Here, we used high resolution nanoscale liquid chromatography coupled to tandem mass spectrometry and hybrid fragmentations methods to resolve the site-specific glycosylation of ITGA2. Of the 10 predicted *N*-glycosylation sites, we were able to identify site occupancy from 7 sites across three different ovarian cancer cell lines (Figure 2A). Overall, the identified *N*-glycans were located at the extracellular domain of ITGA2 comprising N-terminal headpiece (α-I domain and β-propeller domain) as well as C-terminal leg regions (thigh and calf domain). The α-I domain of integrin is recognized to mediate collagen binding and undergo conformational changes in response to receptor activation in a cation-dependent manner (Tuckwell et al., 1995). On the other hand, the C-terminal thigh and calf domains are relatively rigid but have similar immunoglobulin-like structures which confer the interdomain flexibility (Campbell and Humphries, 2011). To convey the main characteristics at each site, the abundance of each glycan was quantified using Skyline software and classified into oligomannose-, hybrid-, and complex-type glycans. The representative *N*-glycopeptides site occupancy and HCD MS/MS spectrum is shown to exemplify its identification of glycan composition at site N343 (Figures 2B and S3). The bar graphs summarize diverse classes of glycoforms present at the individual glycosite as well as the predominant structure identified at each site (Figure 2C and Table S1). Three sites (N432, N460 and N1074) mainly contained oligomannose structures ranging from mannose-5 (M5; Man5GlcNAc2) to mannose-9 (M9; Man9GlcNAc2), in contrast sites N343, N475, N699, and N1057 had complex *N*-glycan structures with high abundance of terminal sialylated glycans. In particular, sites N475 and N1057 were predominantly occupied by biantennary sialylated complex type structures (HexNAc(4)Hex(5)Fuc(1)NeuAc(1) or HexNAc(4)Hex(5)Fuc(1)NeuAc(2)) (Figure 2C). Predicted but unoccupied *N*-glycosylation sites were also characterized and quantified; they formed a trivial proportion of the ITGA2 peptides.

In regards to *O*-glycosylation, we detected 9 *O*-glycosylation sites and the MS data was manually checked for site localization and peptide sequence. The *O*-glycans were mainly composed of core 1 or core 2 *O*-glycans with some sites exhibiting the T antigens (Figure 2D and Table S2).

### Site-specific *N*-glycosylation alters the abundance of ITGA2 on the cell surface

**Figure 3:**
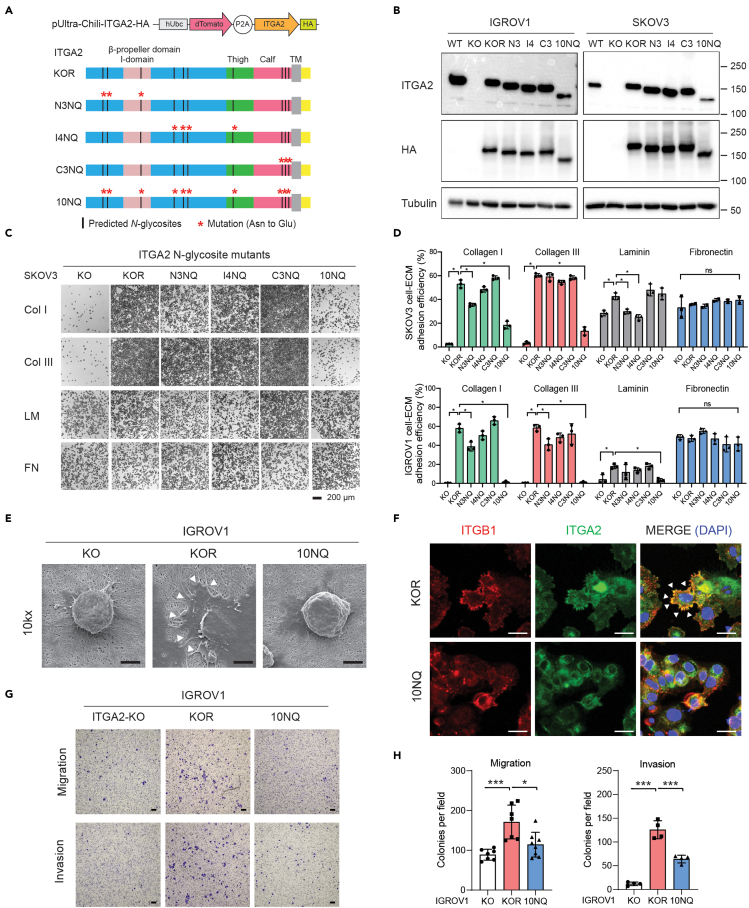
*N*-glycosylation of ITGA2 affects cell-ECM adhesion (A) Schematic representation of ITGA2 *N*-glycosites mutant constructs. Red asterisk indicates amino acid point mutation from Asn to Glu. The constructs are designed in KOR background (ΔITGA2 wildtype rescue), N3NQ (N105Q, N112Q, N343Q), I4NQ (N432Q, N460Q, N475Q, N699Q), C3NQ (N1057Q, N1074Q, N1081Q), and 10NQ (total N-glycosites mutation). (B) Western blot with expression of mutant ITGA2 and C-terminal HA tag with corresponding mass shift. (C) Static cancer cell-ECM adhesion assay. Representative images for ΔITGA2 (KO), KOR, and *N*-glycosite mutants (N3NQ, I4NQ, C3NQ, and 10NQ) cell adhesion to different ECM proteins. (D) Bar charts show the quantification of the percentage of cell adhesion efficiency (mean ± SD) of SKOV3 and IGROV1 cells (ANOVA, ∗p < 0.05). (E) Representative scanning electron microscope images with cellular adherence of IGROV1 KO, KOR, and 10NQ cells on 1.5% collagen-coated inserts. White arrows indicate membrane protrusions. Scale bar 5 μm. (F) Immunofluorescence images of KOR and 10 NQ mutant cells co-stained with anti-HA (ITGA2-HA), Golgi marker (GM130) and nucleus (DAPI). Scale bar 20 μm. (G) Representative 24h cell migration assay through 8 μm hanging inserts and 48h invasion assay with collagen-precoated inserts. Scale bar 100 μm. (H) Bar charts show the mean ± SD of the migrated or invaded cells per field (mean ± SD) (ANOVA, ∗p < 0.05).

Our data suggested that *N*-glycosylation appears persistently on ITGA2 with substantially divergent subtypes of *N*-glycans in a site-specific manner. In order to gain insight into the function of ITGA2 *N*-glycosylation, we site-specifically mutated individual, combination, and all 10 predicted *N*-glycosylation sites to glutamine (Q) in order to abolish *N*-glycosylation of ITGA2. Briefly, the C-terminal fused HA tagged-ITGA2 mutants was built on a lentiviral-based bicistronic vector and transduced into previously established *ΔITGA2* cancer cells (Huang et al., 2020). We classified *N*-glycosite mutant cells into five different groups, KOR (knockout rescue/with intact *N*-glycosylation), N-terminal mutant N3NQ (N105Q, N112Q, N343Q), intermediate mutant I4NQ (N432Q, N460Q, N475Q, N699Q), C-terminal mutant C3NQ (N1057Q, N1074Q, N1081Q), and all sites mutant 10NQ (non *N*-glycosylated ITGA2) (Figure 3A). Substitution of each asparagine to glutamine led to a reduction in *N*-glycans compared to wildtype and KOR ITGA2 as shown by a mobility shift in SDS-PAGE (Figure 3B) and confirmed by LC-MS/MS (Figure S4A). As expected, *N*-glycosylation was completely ablated in ITGA2 10NQ as indicated by no detectable mass change following PNGaseF treatment (Figure S4B).

Interestingly, the expression of ITGA2 and C-terminal fused HA tag in total cell lysates of glycosite mutant cells were not significantly changed, except in 10NQ cells (Figure 3B). Thus, we determined whether *N*-glycosite mutation affects the membranous expression of ITGA2 by flow cytometry. We found ∼15% reduced membranous ITGA2 expression in N3NQ and 40–60% reduction in 10NQ, but only marginal changes in I4NQ and C3NQ mutants in both cell lines (FiguresS5A–S5C). However, we could not exclude the possibility that ITGA2 *N*-glycosylation alters antibody affinity to the corresponding epitope. Additionally, we investigated the other known major collagen-binding integrin subunit ITGA1 which remained unchanged in all the investigated ITGA2 mutant cells indicating that mutated site-specific ITGA2 glycosylation does not affect the expression of ITGA1 as well as its heterodimeric partner β1 integrin (ITGB1) on the cell surface (FiguresS5A–S5C). Together, these results suggested that genetic mutation in the C-terminal leg domain (I4NQ and C3NQ) has little impact in regulating the abundance of ITGA2 expression. However, the mutation in the α-I domain (N3NQ) and even more pronounced 10NQ, reduced ITGA2 expression on the surface of cancer cells.

### ITGA2 glycosylation triggers binding to different extracellular matrix proteins

Receptor conformational changes by amino acid side chain and glycosylation have been recognized as an important mechanism for controlling the affinity of ligand binding by integrins (Luo et al., 2003). We were interested to know whether site-directed *N*-glycan mutants affect specific cell-ECM adhesion. We performed *in vitro* cell adhesion assay on top of different ECM-coated (collagen type I, III, laminin, and fibronectin) plates. Consistent with our previous finding, loss of ITGA2 completely abolished cancer cell adhesion to collagen type I and III but not fibronectin. On the other hand, re-expression of wildtype ITGA2 in the knockout cells (KOR; Knockout Rescue) consistently restored the cell adhesion to collagen I and III and, less strikingly, to laminin (Figure 3C). Despite no significant reduction of surface ITGA2 expression in the N3NQ mutant, cell adhesion to collagen I was markedly reduced. However, the alteration of mutant cell adhesion to collagen type III and laminin seems to be cell line-dependent (Figure 3D). This is likely due to the variability of ligand affinity or redundancy of different integrin subunits among different cancer cell lines. Surprisingly, even though around 50% of non-glycosylated ITGA2 (10NQ) remained expressed on the cell surface, 10NQ mutant cells failed to adhere to the selected known ITGA2 ligands including collagen I, III, and laminin in a cell line-independent manner suggesting that *N*-glycosylation is essential for functional ITGA2-ECM adhesion.

To further investigate glycosylation-dependent cell adhesion to collagen, we visualized cells on collagen by scanning electron microscopy. In consistency with our previous finding, IGROV1 KO cells were unable to adhere to the collagen layer at 6 h. However, re-expression of wildtype ITGA2 restored adhesion property as indicated by a flattened cell surface and formation of obvious membrane protrusions (Figure 3E). Interestingly, loss of *N*-glycosylation limited 10NQ cells' binding capacity to collagen indicating the importance of *N*-glycans in mediating ITGA2-collagen interaction thereby confirming our *in vitro* cell-ECM adhesion assays. The functional α2β1 integrin heterodimer is essential to form the subcellular adhesion foci and the downstream focal adhesion signaling (Michael and Parsons, 2020). As a result, we analyzed the cellular localization of the integrin heterodimer on collagen-coated slides by immunofluorescence. We identified a significant colocalization of α2β1 integrin at the membrane protrusions of KOR but not in 10NQ cells in which the integrin signal is rather dispersed throughout the cell (Figure 3F). Furthermore, we evaluated the *in vitro* cell migration and invasion ability of ITGA2-KO, KOR and 10NQ cells (Figures 3G and 3H). The re-overexpression of glycosylated-ITGA2 showed enhanced cell migration and invasion capability compared to KO and mutant 10NQ cells, suggesting that ITGA2-specific *N*-glycosylation may be involved in cancer cell-adherence to distant sites. A similar trend was found for ITGA2-edited OVCAR4 cell lines harboring *TP53* mutation and are, low in endogenous ITGA2 expression, showing a promoting role of ITGA2 overexpression in collagen-adhesion, cell migration, and collagen-dependent survival (Figures S6A–S6C). In line with IGROV1 and SKOV3, overexpression of mutant ITGA2-10NQ resulted in non-functional integrin mediated adhesion and downstream survival signaling (Figures S6D and S6E). We also noticed a partial accumulation of 10NQ ITGA2 colocalized with the *cis*-Golgi characterized by the marker GM130 (Figure S7) suggesting a possible defect in *N*-glycan biosynthesis of ITGA2 in the canonical protein secretory pathway.

### *N*-glycosylation protects ITGA2 from proteasome degradation

**Figure 4:**
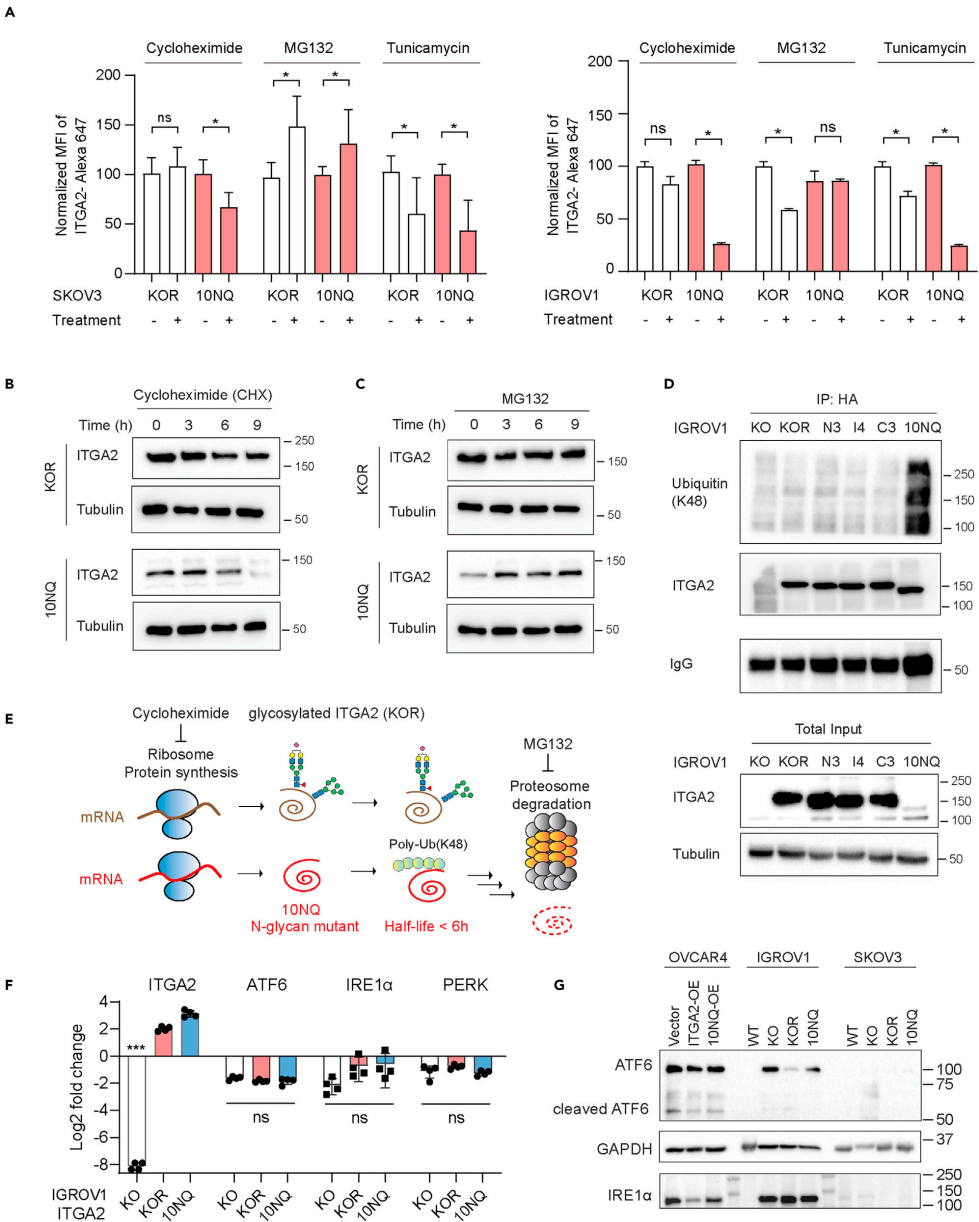
*N*-glycosylation of ITGA2 affects membranous protein stability and turnover (A) Flow cytometry of cancer cell surface ITGA2 expression in KOR and 10NQ cells treated with 2 μM cycloheximide, 10 μM MG132, and 10 μg/mL tunicamycin for 16 h. Bar charts show the quantification (mean ± SD) of the mean fluorescence intensity of ITGA2 normalized to untreated control group (Student t-test ∗p < 0.05). Experiments were performed in independent biological replicates (n = 3). (B and C) IGROV1 KOR, and 10NQ cells were treated with 10 μM MG132 or (C) 2 μM cycloheximide (CHX) as indicated time points 0, 3, 6, and 9 h before harvested protein lysates and subjected to Western blot. (D) Ubiquitination of IGROV1 ITGA2 glycosite mutant was monitored by Western blot. ITGA2 proteins were immunoprecipitated with anti-HA antibody and then immunoblotted with K48-polyubiquitin antibody. (E) Schematic presentation of glycosylated and non-glycosylated ITGA2 synthesis and degradation pathway. (F) RT-qPCR analysis of ER/UPR stress related genes in IGROV1 KO, KOR and 10NQ cells. The Log2 fold change of gene expression was normalized to IGROV1 WT cells.(ANOVA ∗∗∗p < 0.001). (G) ATF6 and IRE1α expression in ITGA2 wildtype and 10NQ overexpression cells analyzed by Western blot analysis.

As the level of cell surface ITGA2 was found to be significantly higher in KOR than non-glycosylated 10NQ, we investigated whether *N*-glycosylation affects ITGA2 stability and translocation to the membrane. The abundance of membranous ITGA2 was determined in the presence of protein synthesis inhibitor cycloheximide (CHX), proteasome inhibitor (MG132), and tunicamycin (*N*-glycosylation inhibitor) using flow cytometry. A dramatic reduction of membranous ITGA2 in the presence of CHX was observed for 10NQ cells (Figure 4A). Blocking the *N*-linked glycosylation pathway by tunicamycin significantly reduced membranous ITGA2 in both KOR and 10NQ cells. This supports the idea that the impaired *N*-glycosylation network affects the biosynthesis of ITGA2. Moreover, the half-life of 10NQ and wildtype ITGA2 was investigated in the presence of CHX. The protein turnover for 10NQ appeared faster compared to wildtype ITGA2 in both dose- and time-dependent manner as shown by a dramatic loss of ITGA2 expression in 10NQ cells (Figures 4B and S8). On the other hand, increased accumulation of ITGA2 in 10NQ cells treated with the 26S proteasome inhibitor MG132 was observed suggesting that *N*-glycosylation stabilizes ITGA2 to avoid proteasome-mediated protein degradation (Figure 4C). To identify the underlying mechanism involved in the degradation of non-glycosylated ITGA2, we examined the levels of ubiquitination of ITGA2. Previous studies by Yoshida et al., have demonstrated that the oligomannose type of *N*-glycans on integrin β1 contributes to poly-ubiquitination and hence proteasome degradation through the F-box protein Fbx2 (Yoshida et al., 2002). Mizushima et al., further suggested that the ubiquitin ligase complex primarily recognizes Man(3)GlcNAc(2) structures, thereby explaining the broad substrate specificity to various glycoproteins (Mizushima et al., 2007). These results supported the concept that ubiquitination and the *N*-glycosylation network collaborate in quality control mechanisms of glycoprotein translocation and biosynthesis. In detail, we examined the levels of lysine K48 ubiquitination, a canonical degradation marker, using immunoprecipitated ITGA2 protein lysates from different *N*-glycosite mutant cells. Our results showed that K48-ubiquitination was dramatically accumulated in 10NQ cells compared to wildtype and other glycosite mutants (Figures 4D and 4E).

However, to exclude the possibility that overexpression of mutant ITGA2 (10NQ) resulted in unexpected unfolded protein response (UPR) and endoplasmic reticulum (ER) stress, we assessed the UPR/ER stress-related genes by RT-qPCR. Neither cyclic AMP-dependent transcription factor (ATF-6), protein kinase R-like endoplasmic reticulum kinase (PERK), nor serine/threonine-protein kinase/endoribonuclease (IRE1α) revealed a significant upregulation by comparing KO, KOR, and 10NQ in IGROV1 (Figure 4F). Similar findings were also confirmed in the SKOV3 and OVCAR4 ITGA2-edited cell line (Figure S9A). Moreover, protein expression of cleaved and full-length ATF-6 and IRE1α were analyzed by Western blot in all cell lines investigated (Figure 4G). These results suggested that overexpression of ITGA2 and glycosite-mutant 10NQ ITGA2 has only marginal impact on ER/UPR stress signaling. We also reanalyzed our proteomic data and focused on the KEGG hsa04141:protein processing in ER pathway (Figure S9B).

### Abolished ITGA2 *N*-glycosylation induces apoptosis and collagen organization signaling

**Figure 5:**
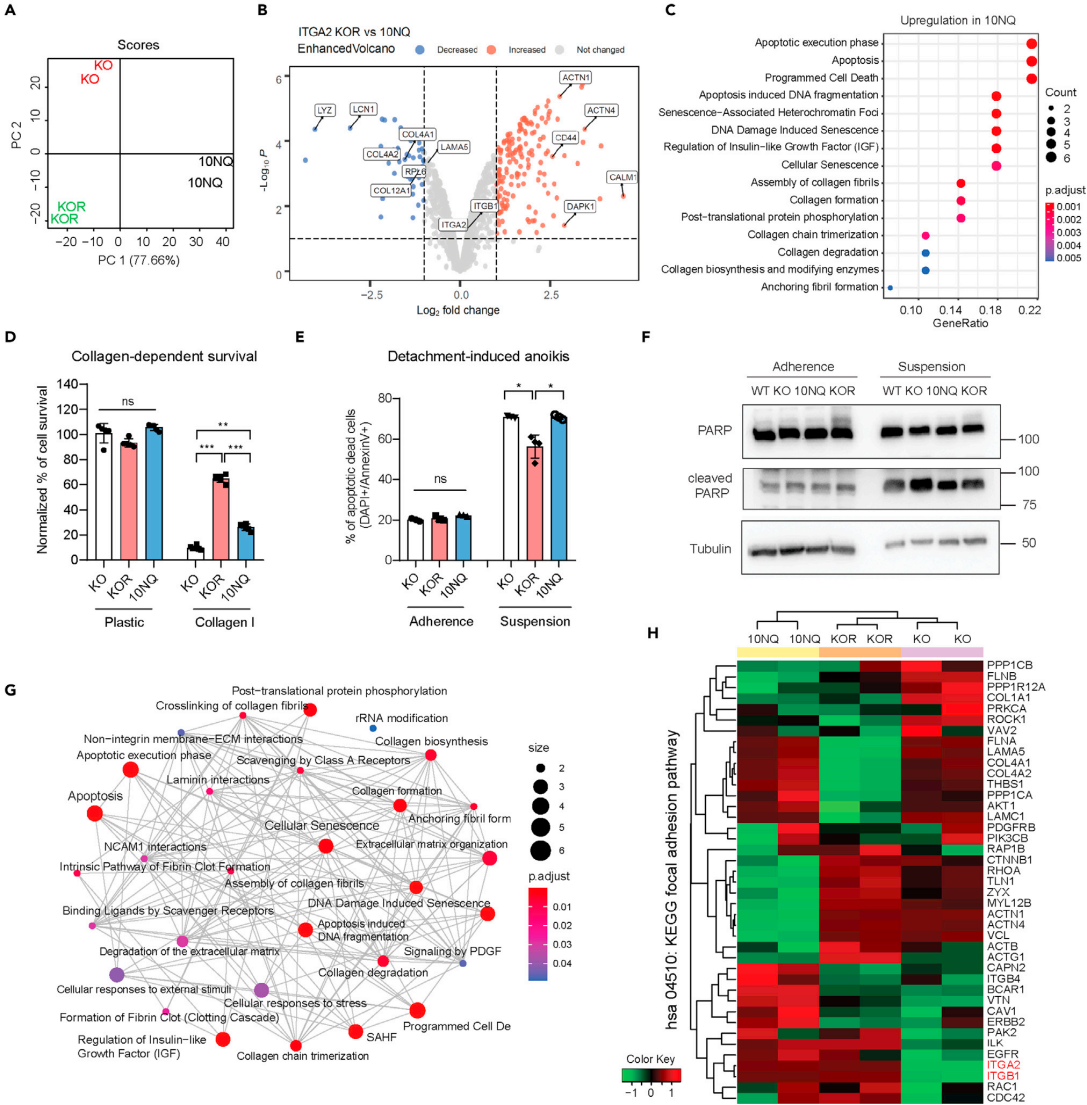
Proteomics identify *N*-glycan dependent ITGA2 signaling (A) Principal component analysis shows distinct clustering of ITGA2 knockout (KO), rescue (KOR) and 10NQ mutant cells. (B) Volcano plot with differentially expressed proteins between KOR and non-glycosylated 10NQ cells, y axis defines the statistical significance -Log10p < 2 and x axis magnitude of Log2-fold change >1 or < −1. Colored circle defines significantly decreased (blue) or increased (red) proteins in KOR cells. (C) Gene set enrichment analysis (GSEA) identified upregulated reactome pathways comparing KOR and N-glycosylated mutant 10NQ cells. The ClusterProfiler dot plot visualization shows enriched terms as dots. The highest ranking of the significantly enriched pathway is displayed. (D) Collagen-dependent cell survival was determined by MTT assay. Equal amounts of KO, KOR, and 10NQ cells were seeded for 48 h cultivation in culture plate or collagen I coated plate. (E) Cell detachment-induced anoikis assay. KO, KOR and 10NQ cells were stained with Annexin V and DAPI after 3 days of cultivation in ultra-low attachment plates to identify apoptotic and dead cells (AnnexinV+/DAPI+). Mean ± SD (∗p < 0.05). (F) Western blot shows increased cleaved PARP in the KO and 10NQ cells in suspension condition. (G) Enrichment map of the interrelation of the top 30 enriched biological processes using ClusterProfiler. (H) Heatmap analysis of KEGG hsa04510:focal adhesion pathway.

In order to elucidate the molecular mechanism following glycosylation-dependent ITGA2 signaling, we analyzed the ITGA2-associated proteome of KO, KOR, and 10NQ cells. Principal component analysis separated all three groups from each other with 77.6% of the total variation accounting for the principal component 1 reflecting the proteomic variability of distinct cellular effects through ITGA2-dependent *N*-glycosylation (Figure 5A). The proteomic analysis identified 1494 proteins in total with 44 significantly downregulated and 156 upregulated proteins in KOR *versus* 10NQ (Figure 5B). We also found that the top enriched biological processes in the 10NQ cells were related to apoptosis, cellular senescence, collagen organization, and ECM-biosynthesis pathway by applying the gene ontological (GO) analysis (Figure 5C). Here, upregulation of collagen formation and ECM organization pathway in 10NQ cells might be a compensatory mechanism due to loss of its receptor-ligand affinity. This is in line with a previous finding reporting that ITGA2 regulates the transcription of fibrillar collagens (Ivaska et al., 1999). Interestingly, we identified not only upregulation of COL4A1, COL4A2, COL5A1, COL12A1 but also an increased expression of laminin subunit alpha 5 (LAMA5) and gamma-1 (LAMC1) in 10NQ cells, suggesting the reorganization of self-ECM components of cancer cells in response to altered integrin expression and *N*-glycosylation.

Since integrin-ligand mediated interaction has been shown to facilitate cancer cell survival signaling (Desgrosellier and Cheresh, 2010), we examined the collagen-dependent cell proliferation among KO, KOR, and 10NQ cells. As expected, reduced cell proliferation was shown in both KO and 10NQ cells only on collagen-coated plates but not on standard cell culture plastic plates (Figure 5D). Further investigation of cell detachment-induced anoikis, a critical step of tumor cells with enhanced mesenchymal features or undergoing malignant transformation, showed a significant increase of apoptotic (Annexin V+/DAPI-)) and dead (Annexin V+/DAPI+) cells in KO and 10NQ cells (Figure 5E). Such enhanced apoptosis in KO and 10NQ cells under suspension condition coincided with elevated levels of cleaved PARP, suggesting that glycosylated ITGA2 promotes anoikis resistance and increases cell survival (Figure 5F). Moreover, the increased release of cellular integrity-associated proteins including actin, alpha-tubulin, and lamin was also identified in the proteomic analysis (Table S3). Overall, the interaction of top enriched biological processes as shown in the enrichment map reflected a robust interplay between integrin, collagen reorganization, and apoptosis-related signaling (Figure 5G).

In regards to the effect of glycosylation on integrin-dependent downstream signaling, it is reasonable to hypothesize that large glycoproteins may sterically restrict efficient integrin–matrix engagement due to increased curvature between the plasma membrane and ECM. However, a previous study sophisticatedly demonstrated that whereas large glycoproteins reduce the overall integrin-binding rate, they significantly enhanced clustering of integrins into focal adhesions through a physical kinetic trap (Paszek et al., 2014). This result suggested that membrane glycocalyx can promote integrin clustering, facilitate focal adhesion assembly, and integrin-dependent growth factor signaling to support cell survival. Therefore, we examined the differential focal adhesion protein expression comparing our ITGA2-KO, KOR, and 10NQ cells. Unsupervised heatmap clustering (Figure 5H) and KEGG signaling pathview (Figure S10) both revealed downregulated focal adhesion-associated cytoskeleton proteins (actinin, talin, vinculin, zyxin) in non-glycosylated 10NQ cells compared to KOR, suggesting that impaired cell growth and survival signal may reflect the downregulation of ITGA2-dependent focal adhesion axis.

### Alpha 2,6-sialylated ITGA2 fosters cell adhesion

**Figure 6:**
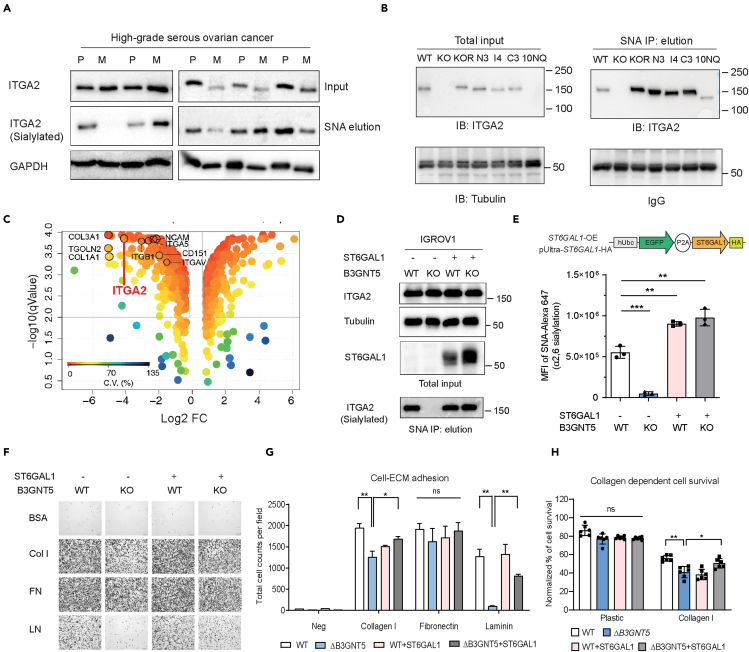
α 2,6-sialylation of ITGA2 promotes cancer cell adhesion to collagen (A and B) Protein lysates from patient-derived tumor samples (n = 5) and (B) glycosylated mutant ITGA2 cell lines were pull down by Sambucus nigra agglutinin (SNA) lectin and blotted with anti-ITGA2 antibody. P, primary tumor, M: metastatic tumor. (C) SNA lectin enrichment combined proteomic analysis identified downregulation of sialylated integrins (α2, α5, αV, and β1) in ΔB3GNT5 cells. (D) SNA lectin pull-down identified loss of ITGA2 sialylation in ΔB3GNT5 cells, and re-expressed ST6GAL1 restored ITGA2 sialylation. (E) Cell surface sialylation was measured by flow cytometry using primary biotinylated-SNA lectin and secondary antibody with Streptavidin-Alexa 647. Bar chart represents the median fluorescence intensity (MFI) of positive SNA epitope (α2,6 sialylation) from three independent experiments (ANOVA ∗∗p < 0.01, ∗∗∗p <0.001). (F) Cell-ECM adhesion assay of WT, ΔB3GNT5 and ST6GAL1-overexpressed IGROV1 cells. (G) Bar chart shows quantification of total adhesion cell numbers mean ± SD from two independent experiment (ANOVA, ∗p < 0.05). (H) Collagen-dependent cell survival was determined by MTT assay after 48h cultivation in culture plastic plate or collagen I coated plate.

The importance of integrin sialylation has been associated with enhanced invasiveness and metastatic potential in multiple cancers (Dobie and Skropeta, 2021). Our glycoproteomic discovery approach identified five *N*-glycosites carrying sialylated glycans (Figure 2C). Therefore, we evaluated the impact of ITGA2 sialylation in the context of ovarian cancer by examining sialylated protein expression in human-derived primary ovarian and metastatic tumors. Using *Sambucus Nigra* agglutinin (SNA) pulldown assay, which preferentially precipitates α2-6 sialylated glycoproteins, we found sialylated ITGA2 present in nearly all of our investigated tissue samples (9 out of 10) (Figure 6A). Furthermore, we investigated whether sialylation is also enriched in certain structural domains of ITGA2 using our established mutant cell lines (N3NQ, I4NQ, C3NQ, and 10NQ). We found that protein sialylation is ubiquitously distributed throughout the entire ITGA2 spectrum of *N*-glycosite mutants (Figure 6B). Considering the abundance of α2-6 sialylation on integrin, it is important to verify which glycosyltransferases are involved in this process. We recently identified beta-galactoside alpha-2,6-sialyltransferase 1 (*ST6GAL1*) among all human sialyltransferases being silenced upon glycosphingolipid-encoding gene editing of Beta-1,3-N-acetyl-glucosaminyl-transferase 5, *B3GNT5* (Alam et al., 2017). Thus, we subjected lysates of those cells to immunoprecipitation using SNA lectin combined with LC–MS/MS analysis using a LTQ-Orbitrap mass spectrometer. Proteomic analysis identified a total of 1118 proteins and 179 proteins were significantly downregulated in *ΔB3GNT5* cells (log2 FC < −2; adjust -log q value > 2) (Figure 6C). Among the identified proteins, integrin α2, α5, αV, and β1 subunits and their ECM ligands were significantly reduced in *ΔB3GNT5* cells suggesting that those integrins are potentially sialylated by ST6GAL1 in wildtype cells (Figure 6C). The reduction of sialylated ITGA2 in *ΔB3GNT5* cells was further validated using SNA lectin pulldown combined with Western blot analysis (Figure 6D). Constitutive expression of ST6GAL1 in the *ΔB3GNT5* cells not only restored cell surface sialylation but also specifically on ITGA2 (Figures 6D and 6E). We next examined the cell-ECM adhesion using our *ΔB3GNT5* cells in combination with ST6GAL1 overexpression. As expected, we identified a significant reduction of cell adhesion to collagen I and laminin but not fibronectin confirming that increased ITGA2 sialylation promotes enhanced integrin-dependent cell-ECM adhesion (Figures 6F and 6G). Moreover, the effect of ST6GAL1 re-expression in *ΔB3GNT5* cells increased cell surface α2,6 sialylation and promoted ITGA2-mediated collagen-dependent survival (Figure 6H). In order to generalize the role of sialylation in mediating ITGA2 signaling, we generated ST6GAL1, ST6GAL1+ITGA2 (DOE) dual-overexpressed OVCAR4 cell lines (Figures S11A and S11B). Despite a remarkable increase of 2,6 sialylation in ST6GAL1-OE cells, cell adhesion to collagen was only marginally increased due to endogenous low levels of ITGA2 in OVCAR4. On the other hand, dual overexpression of ST6GAL1 and ITGA2 significantly enhanced cell-collagen adhesion (Figure S11C) and collagen-dependent survival as compared to vector control (Figure S11D). Taken together, our findings support the hypothesis that apart from overall site-specific *N*-glycosylation, α2-6 sialylation participates in facilitating ITGA2-dependent cancer cell survival.

## Discussion

In summary, our in-depth glycoproteomic analysis offers a detailed and site-specific glycosylation signature of human ITGA2 in ovarian cancer cells. We provide for the first time a detailed map of human ITGA2 glycosylation derived from cancer cells. In particular for *N*-glycans, we identify 7 sites occupied with substantially divergent *N*-linked glycans with predominant complex and oligomannose type glycans in our cell lines. Among the 10 predicted *N*-glycosylation sites of ITGA2, mutation of each individual site has a marginal effect on ITGA2 abundance. Nevertheless, in *N*-glycosylation-depleted 10NQ cells shared a similar phenotype as *ΔITGA2* cells in reducing collagen-dependent cell adhesion and survival. Mechanistically, abolished *N*-glycosylation results in destabilization and proteasome degradation of ITGA2 through poly-ubiquitination. Proteomic analysis of cells harboring wildtype and glycosite mutant ITGA2 revealed increased cellular apoptosis, collagen reorganization, and impaired focal adhesion signaling upon loss of *N*-glycans. Overall, our results highlight the importance of total *N*-glycosylation and, in particular, sialylation in regulating ECM-integrin interaction as well as altered cellular characteristics in cancer cells.

The aberrant glycosylation of integrins have been implicated in multiple cancers (Marsico et al., 2018), however, natural variants that cause loss or gain of function due to individual glycosylation sites have not been reported for ITGA2 so far. Despite the fact that the crystal structure of a complete integrin-ligand complex has not been reported, the interaction of recombinant α2-I domain (from 172 to 355 aa) with collagen peptides GFOGER suggested three major loops which coordinate a divalent cation to engage collagen binding (Emsley et al., 2000). Based on the current structural model, the three N-terminal glycosites are not proximal to the collagen-binding interface. However, the large *N*-glycans within the α-I domain could potentially regulate conformational change and ligand binding allosterically, unlike other α-leg domains which have relatively rigid structures (Campbell and Humphries, 2011). The critical role of *N*-glycans in regulating integrin ligand affinity was also previously demonstrated for β subunits. Introducing an *N*-glycosylation sequence (N303-I304-T305) located at the hybrid-I-like domain on β1 or β3 integrin stabilized the open conformation of integrins and activated the metal ion-dependent binding site, thereby increasing their affinity for ligands (Luo et al., 2003). More recently, removal of *N*-glycosylation sites on the I-like domain of integrin β1 (N212, N269, and N363) suppressed focal adhesion kinase signaling and cell migration in both donor and recipient cells through extracellular vesicles (Cao et al., 2021). As a result, we were interested to know whether modulation of *N*-glycosylation sites on α2 subunit may affect ligand binding affinity, heterodimeric formation of α2β1 integrin, and downstream signaling. Our data revealed that the N-terminal glycosite (N105, N112, and N343) in the α-I domain plays a pivotal role in collagen and laminin binding. Removal of total *N*-glycans showed downregulation of focal adhesion signaling and increased cellular apoptosis. Interestingly, despite no significant reduction of total and membranous β1 integrin expression in non-glycosylated 10NQ cells (Table S3 and Figure S5), the collagen-dependent colocalization of α2β1 heterodimer at the adhesion foci is evidently lost. This is likely due to the constitutively expressed β1 integrin which is also required to form functional heterodimers with at least 12 reported α subunits (Campbell and Humphries, 2011).

Li et al. have demonstrated that *N*-glycans and the lower integrin leg domains regulate α5β1 integrin activity allosterically using structurally well-characterized α5β1 (Li et al., 2017). Complex *N*-glycans stabilized the extended active conformation raising ligand affinity for α5β1 ectodomain by 8- to 10-fold as compared to truncated glycans. It was also proposed that the larger *N*-glycans might exert repulsive interaction within the domain interfaces. However, the function of each individual *N*-glycan site of α5β1 or other integrins has not been documented. So far, only a few studies have investigated the biological and structural functions of *N*-glycosylation sites of α2β1 integrin despite its association to multiple malignancies. A recent study systematically identified HepG2 liver cancer cell surface *N*-glycoproteins by combining metabolic sugar labeling, copper-free click chemistry, and MS-based proteomics (Xiao et al., 2016). A total of 280 unique *N*-glycosylated sites from 168 surface proteins were identified, including seven ITGA2 *N*-glycosylation sites (N105, N112, N343, N432, N460, N1057, and N1074) with three of them being sialylated (N105, N343, and N1057). Our ITGA2 site-specific determination of total glycosylation revealed also 7 *N*-glycosylation sites and 5 sialylation sites (N343, N460, N475, N699, and N1057) spanning the entire structural domains of ITGA2 with complex type sialylated glycans in human ovarian cancer cells (Figure 2C). In agreement with our finding, the importance of sialylation on integrins has been associated with enhanced cell adhesion, invasiveness, and metastatic potential in multiple cancers (Dobie and Skropeta, 2021). Increased sialylation of integrin β1 through alpha-2,6-sialyltransferase 1 (ST6GAL1) in colon cancer cells was associated with cancer progression by promoting cell motility and attachment to collagen I and laminin (Seales et al., 2005). Similarly, elevated sialylation of integrin α6 enhances integrin-mediated cell mobility on collagen and fibronectin in pancreatic cancer cells (Almaraz et al., 2012). More recently, concomitant upregulation of ITGA2 and increased levels of sialylated bi- and tri- antennary *N*-glycans was correlated with metastatic potential in breast cancer cell lines using glycomics (Peng et al., 2019). It is also reported that α2,6 sialylation is required for the activation of integrin β1 and facilitate its interaction with EGFR, a receptor tyrosine kinase that mediates cell migration and survival signaling (Hou et al., 2016). In contrast, Yamamoto et al. suggested that increasing α2,6 sialylation abolished cell invasion and cell adhesion to collagen and fibronectin whereas increasing α2,3 sialylation stimulates invasiveness of glioma cells (Yamamoto et al., 2001). Disruption of glioma cell-ECM interaction reduced focal adhesion signaling *in vitro* and tumor formation *in vivo*. Although the authors could not exclude the possibility that alteration of sialic acid on other cell surface proteins is at least in part attributed from α3β1 integrin expressed in these cells. Similarly, α2,6 sialylation of α2β1 and α5β1 integrins impaired adhesion on collagen IV using breast cancer cell line MDA-MB-231 (Yuan et al., 2016). Despite contrary findings, the importance of protein sialylation in modulating integrin-mediated cancer cell adhesion and migration is evident; however, tumor-associated cellular characteristics seem to depend on tissue-specific expression of heterodimeric integrins and their interaction with environmental ECM ligands.

Apart from the *N*-glycans reported herein, our comprehensive glycoproteomic analysis also revealed various *O*-GalNAc-sites on ITGA2. Although we did not provide functional evidence of site-specific *O*-glycosylation in regulating ITGA2 activity, several studies associated the *O*-glycosylated integrin α2β1 with a more aggressive malignant phenotype. For instance, overexpression of core 1 β1,3-galactosyltransferase (C1GALT1) modifying the core 1 *O*-glycans on β1 integrin increased cell adhesion to ECM coinciding with an advanced tumor stage and poor survival in hepatocellular carcinoma (Liu et al., 2014). By contrast, the β3-N-acetylglucosaminyltransferase-6 (B3GNT6) modifies core 3 O-glycan leading to decreased levels of the α2β1 integrin, decreased activation of focal adhesion kinase and lamellipodia formation which, in turn, suppresses tumor formation and metastasis of prostate carcinoma (Lee et al., 2009). However, due to high abundance of *O*-glycosylation sites on integrin, whether site- and structure- specific *O*-glycans affects ITGA2 protein stability and ligand affinity in cancer cells needs to be systematically addressed similar to what has been done with our *N*-glycan mutagenesis approach or through the simple cell technology (Steentoft et al., 2013).

In addition to integrin glycosylation itself, integrin ligands, such as collagens, laminins, and fibronectins as well as glycosaminoglycans and glycosphingolipids also play critical roles in mediating integrin functions (Marsico et al., 2018). In particular, carbohydrate-to-carbohydrate interaction by ganglioside GM3 is in synergy with integrin-dependent adhesion (Hakomori, 2004). In contrast, sialylated ganglioside GT1b and GD3 interact with oligomannose residues on the α5 integrin, inhibiting α5β1-mediated cell adhesion of keratinocytes to fibronectin (Wang et al., 2001). Additionally, cell surface α2,3-sialylation on integrin α2 is required for integrin-dependent cell adhesion to collagen type I and the interaction with asialo-GM1 in prostate cancer (Van Slambrouck et al., 2014). Likewise, we also previously showed that alteration of the neo-lacto series glycosphingolipids biosynthesis pathway coincides with reduction of α2-6 sialylation on *N*-glycoproteins, in particular affecting ITGA2-mediated cell adhesion to collagen and laminin (Alam et al., 2017) (Figure 6F).

Overall, accumulating evidence suggests that not only *N*- or *O*-linked glycosylation, but also glycosphingolipids may be involved in regulating the functionality and ligand binding affinity of integrins which may direct cell fate towards a malignant phenotype. Although MS-based glycomics provide the possibility to analyze the *N*-glycomes, identification of site-specific glycopeptides remains challenging due to difficulties in dissociation methods and effective enrichment and separation methods. Moreover, the complexity and the dynamic interaction of glycosylation networks between integrins, ECM, and the associated membrane glycoconjugates remain to be elucidated. The increasing number and further development of glycoproteomic and computational analysis will continue to help us better understand how site-specific integrin glycosylation mediates its ligand interaction and subsequent signaling. Our generated glycoproteomic profile of ITGA2 from cancer cells may be useful to serve as a MS-library for identification of certain glycopeptide signatures in clinically relevant samples in future.

### Limitations of the study

In this study, we have used site-specific glycosylation analysis and functional glycomics to provide an insight into the role of human ITGA2 glycosylation in protein stability and collagen binding affinity. We acknowledge that our experimental strategy (domain-specific mutational analysis rather than individual sites) and biochemical tools utilized lacks direct evidence to prove that a particular glycan structure at a site of ITGA2 contributes to a specific function, e.g., glycan moiety interacting with ligand or conformational change affected by altered glycosylation. In addition, we have also functionally overlooked the potential of O-glycans that may contribute to ITGA2 activity. Moreover, glycoproteomic experiments have been performed in cancer cells only and our ITGA2-glycosylation data are therefore not necessarily reflective of ITGA2 glycosylation from “normal” cells.

## STAR★Methods

### Key resource table

**Table:** 

REAGENT or RESOURCE	SOURCE	IDENTIFIER
**Antibodies**		

Integrin β1 (D2E5)	Cell Signaling Technology	Cat#9699; RRID:AB_11178800
Integrin β1 (12G10)	Abcam	Catab30394
Integrin β1 (CD29)-BV510	BD Biosciences	Cat#747747; RRID:AB_2868388
Integrin α1 (CD49a) Alexa 647	Biolegend	Cat328310; RRID:AB_2129242
Integrin α2	Abcam	Catab181548; RRID:AB_2847852
Integrin α2 (CD49b)-FITC	BD Biosciences	Cat555498; RRID:AB_395888
Integrin α2 (CD49b)-Alexa 647	BD Biosciences	Cat564314; RRID:AB_2738739
E-Cadherin (24E10)	Cell Signaling Technology	Cat3195; RRID:AB_2291471
K48-linkage Specific Polyubiquitin (D9D5)	Cell Signaling Technology	Cat4289; RRID:AB_10557239
HA-Tag	Cell Signaling Technology	Cat3724; RRID:AB_1549585
AnnexinV-FITC	Biolegend	640906
Tubulin	Cell Signaling Technology	Cat2148; RRID:AB_2288042
GAPDH	Santa Cruz	Catsc47724; RRID:AB_627678
PARP	Cell Signaling Technology	Cat9542; RRID:AB_2160739
Cleaved PARP	Cell Signaling Technology	Cat9541; RRID:AB_331426
ATF-6	Cell Signaling Technology	Cat65880; RRID:AB_2799696
IRE1α	Cell Signaling Technology	Cat3294; RRID:AB_823545
Actin	Sigma-Aldrich	CatA5441; RRID:AB_476744
SignalStain®Boost IHC Detection Reagent (HRP, Mouse)	Cell Signaling Technology	Cat8125; RRID:AB_10547893
SignalStain®Boost IHC Detection Reagent (HRP, Rabbit)	Cell Signaling Technology	Cat8114; RRID:AB_10544930
Anti-mouse IgG, HRP-linked Antibody	Cell Signaling Technology	Cat7076; RRID:AB_330924
Anti-rabbit IgG, HRP-linked Antibody	Cell Signaling Technology	Cat7074; RRID:AB_2099233
Sambucus Nigra Lectin (Agarose bound)	Vector Laboratories	AL-1303-2

**Chemicals, peptides, and recombinant proteins**		

Collagen type I (from Human placenta)	Sigma-Aldrich	C7774
Collagen type III (From human placenta)	Advanced BioMatrix	5019
Collagen type IV (From Human Cell)	Sigma-Aldrich	C7521
Fibronectin from Human Plasma	Millipore	FC010
Laminin (From EHS murine sarcoma)	Sigma-Aldrich	L2020
DAPI	BD Biosciences	564907
FITC Annexin V Apoptosis Detection Kit I	BD Biosciences	556547
Pierce™ BCA Protein Assay Kit	ThermoFisher Scientific	23225
Diaminobenzidine substrate kit (DAB)	ThermoFisher Scientific	34002
ProLong® Gold antifade reagent	Cell Signaling Technology	9071
Radioimmunoprecipitation assay buffer (RIPA)	Cell Signaling Technology	9806
Thiazolyl Blue Tetrazolium Bromide (MTT)	Sigma-Aldrich	M2128
Agarose, low gelling temperature	Sigma-Aldrich	A9414
Fetal Bovine Serum	Sigma-Aldrich	F7524
Bovine serum albumin	Sigma-Aldrich	A9418
Proteinase Inhibitor Cocktails	Sigma-Aldrich	P8340
Cell-dissociation solution non-enzymatic	Sigma-Aldrich	C5914
MG132	Sigma-Aldrich	M8699
Cycloheximide	Sigma-Aldrich	C4859
Tunicamycin	Sigma-Aldrich	T7765
pGEM®-T Easy Vector System	Promega	A1360
Wizard® SV Gel and PCR Clean-Up System	Promega	A9281
Pfu DNA Polymerase	Promega	M7741
Sequencing-grade modified trypsin	Promega	V5111
jetPEI DNA transfection reagent	Polyplus-transfection	101-10N
Trans IT-X2 transfection reagent	Mirus	MIR6004
Q5 Site-Directed Mutagenesis Kit	New England Biolabs	E0554S
PNGase F	New England Biolabs	P0708L

**Experimental models: Cell lines**		

IGROV1	ATCC	RRID:CVCL_1304
SK-OV-3	ATCC	RRID:CVCL_0532
OVCAR3	ATCC	RRID:CVCL_0465
OVCAR4	Oncotest GmbH	RRID:CVCL_1627
OVCAR5	Oncotest GmbH	RRID:CVCL_1628
OVCAR8	Oncotest GmbH	RRID:CVCL_1629
CAOV3	ATCC	RRID:CVCL_0201
BG1	Oncotest GmbH	RRID:CVCL_6571
SKOV3.ip1		RRID:CVCL_0C84

**Oligonucleotides**		

ITGA2 N105Q For AAGCATTCCAcaaGTTACTGAGATG	This paper	NA
ITGA2 N105Q Rev GTTGAAGTTTGCAAATTTAGTTTTTC	This paper	NA
ITGA2 N112Q For GATGAAAACCcaaATGAGCCTCG	This paper	NA
ITGA2 N112Q Rev TCAGTAACATTTGGAATGC	This paper	NA
ITGA2 N343Q For ATACTTTTTCcaaGTGTCTGATGAAGC	This paper	NA
ITGA2 N343Q Rev CTTTCTGTTGGAATACTAGC	This paper	NA
ITGA2 N432Q For GCAGGACAGAcaaCACAGTTCATATTTAG	This paper	NA
ITGA2 N432Q Rev AGAATTTGGTCAAAGGCTTG	This paper	NA
ITGA2 N460Q For TCCTCGGGCAcaaTATACCGGCC	This paper	NA
ITGA2 N460Q Rev GCACCAGCAACAAAGTGAG	This paper	NA
ITGA2 N475Q For TGAGAATGGCcagATCACGGTTATTC	This paper	NA
ITGA2 N475Q Rev TTCACACTATATAGCACTATCTGG	This paper	NA
ITGA2 N699Q For CATTGTATATcaaATCACACTTGATGC	This paper	NA
ITGA2 N699Q Rev GCCACTTGATTGTTTTGC	This paper	NA
ITGA2 N1057Q For TTCCTGTAGTcaaGTTACCTGCTG	This paper	NA
ITGA2 N1057Q Rev GCAGTTCTGCAGTTCAATTC	This paper	NA
ITGA2 N1074Q For ATACTTTGTTcaaGTGACTACCAGAATTTG	This paper	NA
ITGa2 N1074Q Rev TCTCCTTTCATGTGAACG	This paper	NA
ITGA2 N1081Q For CAGAATTTGGcaaGGGACTTTCG	This paper	NA
ITGA2 N1081Q Rev GTAGTCACTTGAACAAAGTATTC	This paper	NA
ITGA2 614F Sequencing primer GCCCCACAAAGACACAGGT	This paper	NA
ITGA2 1990F Sequencing primer ACCGATGTGTCTATTGGTGCC	This paper	NA
ITGA2 2003R Sequencing Primer TGGTCAACAAGAATGCTCAGA	This paper	NA
ATF-6 For QPCR TTGACATTTTTGGTCTTGTGG	Balakrishnan et al., 2013	NA
ATF-6 Rev GCAGAAGGGGAGACACATTT	Balakrishnan et al., 2013	NA
PERK For TCATCCAGCCTTAGCAAACC	Balakrishnan et al., 2013	NA
PERK Rev ATGCTTTCACGGTCTTGGTC	Balakrishnan et al., 2013	NA
IRE1a For CTCTGTCCGTACCGCCC	Balakrishnan et al., 2013	NA
IRE1a Rev GAAGCGTCACTGTGCTGGT	Balakrishnan et al., 2013	NA

**Recombinant DNA**		

pUltra	Addgene	24129
pUltra-Chili	Addgene	48687
pCMVR8.74	Addgene	22036
pMD2.G	Addgene	12259
mCherry-Integrin-Alpha2-N-18	Addgene	55063
pUltra-Chili-ITGA2-HA	Huang et al., 2020	NA
pUltra-Chili-ITGA2-HA (KOR)	Huang et al., 2020	NA
pUltra-Chili-ITGA2-N3NQ-HA	This paper	NA
pUltra-Chili-ITGA2-I4NQ-HA	This paper	NA
pUltra-Chili-ITGA2-C3NQ-HA	This paper	NA
pUltra-Chili-ITGA2-10NQ-HA	This paper	NA
pCMV3-ST6GAL1-HA	Sino Biological Inc	HG11590-CY
pUltra-ST6GAL1	This paper	NA

**Software and algorithms**		

R studio 4.0.3	R Core Team, 2017	https://www.R-project.org/
GraphPad Prism 9	Pad Software, Inc.	https://www.graphpad.com/
ImageJ	NIH	https://imagej.nih.gov/ij/
FlowJo v10	Becton Dickinson	https://www.flowjo.com/
Byonic 2.16	Protein Metrics	https://proteinmetrics.com/
Image Lab 6.0.1	Bio-Rad	https://www.bio-rad.com/
Skyline 20.1	MacCoss Lab	https://skyline.ms/
SafeQuant v2.3.4	Ahrne et al., 2016	https://github.com/eahrne/SafeQuant/)
ReactomePA	Yu and He, 2016	NA
Progenesis QI 2.0	Waters	https://www.nonlinear.com/
OMERO v5.6	OME project	https://www.openmicroscopy.org/omero
Adobe illustrator 2021	Adobe	https://www.adobe.com/ch_de/?mv=search&sdid=88X75SKX

### Resource availability

#### Lead contact

Further information and requests for resources should be directed to and will be fulfilled by the lead contact, Francis Jacob (francis.jacob@unibas.ch).

#### Materials availability

All relevant data supporting the key findings of this study are available within the article and its Supplemental Information files or from the corresponding authors upon reasonable request.

### Experimental model and subject details

#### Patient ethics and cell lines

The study was approved by the respective medical ethics committees: The Swiss Medical Ethical Committee (EKNZ 2015-436). A total number of three different ovarian cancer cell lines were purchased via ATCC® and Oncotest GmbH (now CHARLES RIVER LABORATORIES Inc.) maintained in house in RPMI-1640 supplemented with 10% fetal bovine serum (Sigma-Aldrich), 100 U/mL penicillin and 0.1mg/mL streptomycin unless stated differently. All the cell lines were regularly tested for mycoplasma contamination and authenticated using short tandem repeat STR profiling (Microsynth, Switzerland). All cell lines were cultured at 37°C in a 95% humidified atmosphere containing 5% CO_2_.

### Method details

#### Generation of ITGA2-HAKOR(knockout rescue) construct

The human ITGA2 transcript variant 1 open reading frame was amplified using 2U of Pfu DNA polymerase (Promega), 1x Pfu polymerase buffer, 200 μM dNTPs, 300nM primers with XbaI and NheI restriction sites (ITGA2-XbaI_F: gaatctagaATGGGGCCAGAACGGACA and ITGA2-Nhe_R: caagctagcGCTACTGAGCTCTGTGGT), and 30ng cDNA template (Integrin α2-N-18; #addgene 55063) under the following conditions: 95°C for 1 min followed by 30 cycles of 95°C for 30 sec, 55°C for 30 sec, 72°C for 8 min, and finished with 1 cycle at 72°C for 5 min. Amplicons were visualized on 1% agarose gel and purified by Wizard SV gel and PCR Clean-Up System (Promega) and cloning into C-terminal HA tagged pUltra (addgene#24129) or pUltra-Chili (addgene #48687) bicistronic expression vectors. The ligation products were transformed into Stbl3 *E. coli* competent cells and the plasmids were purified and further verified by Sanger DNA sequencing (Microsynth, Switzerland).

#### Generation of ITGA2 *N*-glycosites mutant construct

Q5® Site-Directed Mutagenesis Kit (New England Biolabs, #E0554S) was applied to make site-directed mutagenesis for individuals and a combination of 10 *N*-glycosylation sites as listed below. The mutagenesis primers and PCR amplification condition were designed based on NEBaseChanger v1.2.0 (https://nebasechanger.neb.com/) (Key Resource Table). In general, ten predicted *N*-glycosylation sites (asparagine, N) with encoding sequence AAT or AAC were sequentially mutated to CAA or CAG corresponding to glutamine (Q) in order to generate N3NQ (N105Q, N112Q, N343Q), I4NQ (N432Q, N460Q, N475Q, N699Q), C3NQ (N1057Q, N1074Q, N1081Q), and 10NQ (all 10 predicted sites mutated to Q). PCR amplification with initial denaturation for 30 sec at 98°C, followed by 25 cycles of 98°C for 10 sec, 57-62°C (annealing temperature adjusted for each primer pairs) for 20 sec and 72°C for 7 min, and final extension at 72°C for 2 min. KLD (Kinase, Ligase and DpnI) reaction was performed according to manufacturer’s instructions followed by standard bacterial transformation using Stbl3 *E. coli* competent cells. The plasmids were purified and further verified by Sanger DNA sequencing (Microsynth, Switzerland) before lentiviral transduction.

#### Lentiviral transduction for generating ITGA2 mutant cells

For preparation of lentiviral particles, HEK293T cells were seeded at 50% confluency in a T75 flask one day before transfection. 4 μg of plasmid pUltra-Chili-ITGA2-HA (or ITGA2-mutants) and 2 μg of pMD2.G (Addgene #12259) and 2 μg of pCMVR8.74 (Addgene #22036) were co-transfected using 24 μL of jetPEI reagent in 1 mL of 150 mM NaCl solution (Polyplus-transfection, Chemie Brunschwig AG, Switzerland). Medium was changed 24 h after transfection. Virus supernatant was collected 48 h later and filtered with a 0.45 μm polyvinylidene fluoride filter (Millipore). 3 mL of lentivirus-containing medium was used to transduce previously established Δ*ITGA2* ovarian cancer cells (Huang et al., 2020) to preclude the functional impact of endogenous ITGA2 expression. dTomato + transduced cells were sorted by BD FACS Aria Cell Sorter (BD Bioscience). In addition, pUltra-Chili-ITGA2-HA and pUltra-Chili-10NQ-HA plasmids were used to transduce OVCAR4 cancer cell lines. Plasmid-encoding dTomato + transduced cells were sorted by BD FACS Aria Cell Sorter (BD Bioscience).

#### Generation of ST6GAL1 overexpression cell line

The human ST6GAL1 open reading frame was subcloned from pCMV3-ST6GAL1 (Sino Biological Inc, HG11590-CY) into pUltra (addgene#24129) vector using *XbaI* and *NheI* restriction sites. The ligation products were transformed into *Stbl3 E. Coli* competent cells and the plasmids were purified and further verified by Sanger DNA sequencing (Microsynth, Switzerland). The established pUltra-ST6GAL1 plasmid was used to prepare lentiviral particles as described previously and transduced in OVCAR4 WT, IGROV1 WT and IGROV1 *ΔB3GNT5* cancer cell lines.

#### PNGaseF treatment

PNGaseF enzyme (New England Biolabs, MA, USA, #P0704) treatment was applied to remove the *N*-linked oligosaccharides from glycoproteins. In brief, approximately 20 μg of total protein lysates were diluted with 10X Glycoprotein Denaturing Buffer to make a 10 μL total reaction volume (final concentration 0.5% SDS, 40 mM DTT). Denature glycoprotein by heating at 100°C for 10 min. Make a total reaction volume of 20 μL by adding 2 μL 10XGlycoBuffer 2 (500 mM Sodium Phosphate pH 7.5), 2 μL 10% NP-40 and 6 μL H_2_O. 1 μL of PNGase F were added and mixed gently at 37°C for 1h. The extent of deglycosylation is assessed by mobility shifts on SDS-PAGE and Western blot.

#### Protein stability and turnover assay

To evaluate the role of *N*-glycans on ITGA2 in protein stability, wildtype (WT), ITGA2-knockout-rescue (KOR) and *N*-glycosite mutant (10NQ) cells were treated with 30 μM protein synthesis inhibitor cycloheximide (Sigma) or 1 μM of proteasome inhibitor MG132 (Sigma) at indicated time points (0, 3, 6, 9 h) before harvesting the total cell lysates and subjected to western blot analysis. For cell surface ITGA2 expression analysis, *N*-glycosite mutant cells were treated with cycloheximide, MG132 and tunicamycin independently for 16h, and the single cell suspension was further analyzed by flow cytometry (CytoFlex, Beckman Coulter) using DAPI and Alexa Fluor®647 mouse anti-human ITGA2/CD49b (BD Bioscience, 1:100).

#### Anoikis assay

To evaluate anchorage-independent cell growth, cells were grown in a 96-well ultra-low attachment plate (Corning Costar®) for 3-5 days. Cells were then harvested, washed, and gently dissociated with 0.1% trypsin and stained with a FITC-Annexin V Apoptosis Detection kit (BD Biosciences) according to the manufacturer’s instructions. The percentage of DAPI and FITC positive cells were analyzed by flow cytometry using CytoFLEX (Beckman Coulter).

#### Cell adhesion assay

To evaluate the adhesion properties of cancer cells to ECM proteins, a 96-well Nunc Maxisorp^TM^ flat-bottom plate (Invitrogen) were coated overnight at 4°C using 10 μg/well human type I, III, IV collagen, fibronectin, and Engelbreth-Holm-Swarm tumor (EHS)-derived laminin, which were prepared according to manufacturer’s instructions. The plates were first washed with serum-free RPMI-1640 medium and 0.1% (w/v) BSA to remove unbound proteins, then blocked with serum-free RPMI-1640 medium and 0.5% (w/v) BSA for 45 min at 37°C. EOC cell lines and ascites-derived tumor cells (under passage 0 to 1) were collected and re-suspended in serum-free medium. A total of 5 × 10^4^ cells were seeded in each well in triplicates and incubated for 20 min at 37°C for adhesion. Non-adherent cells were removed by washing twice with serum-free media with 0.1% (w/v) BSA. Adherent cells were fixed with 4% paraformaldehyde solution for 15 min, and stained with 0.05% (w/v) crystal violet for 15 min. Images were acquired using the widefield microscope Olympus IX81, and the number of adherent cells was analyzed using ImageJ software.

#### Cell migration and invasion assay

Approximately 1 × 10^5^ cells were suspended in a serum-free media and seeded into the upper chamber of a 12-well hanging insert with a pore size of 8 μm (Millicell®, Millipore). Cells were incubated at 37°C for 18 h allowing cells to migrate toward the chemo-attractant (RPMI medium containing 10% FBS). After incubation, the medium in the interior part of the insert was removed and the insert immersed in 0.1% crystal violet/4% paraformaldehyde solution for 20 mins. The insert was intensively washed and non-migrated cells were removed from the interior of the insert using a cotton-tip swab. Images were acquired with Nikon ECLIPSE Ti2 and the number of migrated cells were counted from three independent experiments. For invasion assay, (EHS)-derived laminin and collagen I, IV were mixed and pre-coated on the 8 μm polyethylene terephthalate (PET) membrane before seeing the cells.

#### Flow cytometry

Cell-surface integrin expression was analyzed by flow cytometry (CytoFLEX, Beckman Coulter) after antibody labeling. Sub-confluent cancer cell lines or primary ascites-derived tumor cells were harvested, washed, and dissociated using 1X non-enzymatic dissociation buffer (Sigma-Aldrich). Cells were incubated with the following fluorescence-labeled antibodies: Alexa Fluor®647 mouse anti-human ITGA2/CD49b (BD Bioscience, 1:100), FITC-mouse anti-human ITGA2/CD49b (1:100), BV510-mouse anti-human ITGB1/CD29(1:100) at 4°C for 1 h. Matching isotype monoclonal antibodies conjugated to FITC or Alexa Fluor®647 were used as controls (BD Bioscience). All investigated cell lines were gated individually to exclude debris, doublets, or DAPI-staining (BD Bioscience 0.1 μg/mL) to exclude dead cells. Data analysis was performed using FlowJo v10 BD (Becton Dickinson).

#### Immunohistochemistry

Formalin-fixed, paraffin-embedded tissue samples were sectioned with a standard microtome at 3- to 5-μm thickness. After deparaffinization and rehydration, heat-induced (98°C) antigen retrieval was performed in a 10 mM sodium citrate buffer (pH 6.0) at a sub-boiling temperature for 10 min. The slides were incubated with hydrogen peroxide 3% (v/v) for 10 min, washed and blocked with 5% FBS in TBST for 1h at room temperature. Next, slides were incubated with primary antibodies anti-ITGA2 (1:500) (Abcam) at 4°C overnight. Primary antibodies were detected using a SignalStain® Boost IHC anti-rabbit HRP Reagent (Cell signaling Technology). The signal was visualized using a diaminobenzidine substrate kit (DAB, Thermo Fisher Scientific) according to the manufacturer’s instructions and nuclei were counterstained with hematoxylin.

#### Immunofluorescence staining

Ovarian cancer cells were grown on an 8-well tissue culture chamber slides (Sarstedt, Switzerland), fixed with 4% paraformaldehyde, permeabilized with 0.3% Triton X100, and blocked with 5% (w/v) FBS (Sigma-Aldrich), 1% bovine serum albumin (BSA) fraction V, and 0.1% TritonX-100 containing PBS for 1 h at room temperature. Cells were then stained with anti-integrin α2, anti-E-cadherin antibodies for overnight at 4°C. Following extensive washing, corresponding secondary antibodies were added to each chamber and incubated for 2 h. Cells were washed with PBS containing 0.1% Tween 20 and incubated and counterstained with ProLong® Gold antifade reagent with DAPI (Cell Signaling Technology). Fluorescence images were taken by a Zeiss LSM 710 confocal microscope (Zeiss, Feldbach, Switzerland). For patient-derived primary cells, 100-200 μL ascites cell suspension was collected using cytospin for 5 min at 400g on a glass slide. Cells were then fixed with 4% paraformaldehyde, permeabilized, blocked and stained as mentioned above.

#### Immunoblotting

Cells were lysed in 1X radioimmunoprecipitation assay buffer (RIPA, Cell Signaling Technology) containing proteinase inhibitor cocktails (Sigma-Aldrich). Lysates were clarified by centrifugation at 18,000 g for 15 min at 4°C. Clarified lysates were boiled in 1x sample buffer (50 mM Tris-HCl, 1% SDS, 100 mM DTT and 10% glycerol) at 95°C for 5 min and resolved by SDS-PAGE. Proteins were then transferred to a polyvinylidene difluoride (PVDF) membrane (BioRad) and blocked with 5% (w/v) bovine serum albumin in TBST (20mM Tris-Base, 150mM NaCl, pH 7.8, 0.1% Tween 20) for 1 h at room temperature. The membrane was incubated with one of the listed primary antibodies diluted in 5% (w/v) BSA in TBST overnight at 4°C. After extensive washing in TBST, the membrane was incubated with corresponding HRP-conjugated secondary antibodies (1:10,000, Cell Signaling Technology) for 3 h at room temperature. Finally, the membrane was developed using the Super Signal West Dura Extended Duration Substrate (Thermo Fisher Scientific) for detection of HRP. Western blot results were visualized by Gel Doc XR+^TM^ (BioRad) and analyzed by Image Lab^TM^ software (BioRad).

#### Scanning electron microscope

Millicell® cell culture inserts (PICM0RG50, Merck Millipore, Switzerland) were coated with 100 μL 1.5% rat tail collagen type 1 and 4 × 10^4^ IGROV1 ITGA2-KO, KOR and 10NQ cells were added. Cells were allowed to adhere for 3 and 6h before fixed overnight at 4°C using 2.5% glutaraldehyde in 0.03 M K_2_HPO_4_/KH_2_PO_4_ buffer and 2% paraformaldehyde in PBS. Samples were dehydrated using a series of ethanol dilutions (20–100%; 5 min per dilution), and dried with hexamethyldisilazane for 15 min. Samples were sputtered-coated with 3 nm gold and analyzed using a scanning electron microscopy (Tescan Mira3 LMFE, USA).

#### SNA-lectin pull down assay

400 μg of total cell lysates were incubated with 100 μL of agarose-bound Sambucus nigra lectin (SNA, #AL-1303, Vector Laboratories) at 4°C overnight on a rotator. α2,6-sialylated glycoproteins were enriched by centrifugation and washed 3 times with cold lectin buffer (10 mM HEPES, pH 7.5, 0.15 M NaCl, 0.1 mM CaCl_2_, 0.08%, 20 mM lactose). The enriched sialylated glycoproteins were eluted directly by 2X Laemmli SDS buffer and heated at 95°C for 5 min before subjected to SDS-PAGE and western blot analysis.

#### Immunoprecipitation of ITGA2 glycoprotein

Cells were lysed in 1X RIPA assay buffer containing proteinase inhibitor cocktails (Sigma-Aldrich, #P8340). Lysates were clarified by centrifugation at 18,000 g for 15 min at 4°C. The supernatants were subjected to immunoprecipitation with anti-HA magnetic beads (Cell Signaling, #11846) About 750 μg/mL of whole cell lysate was mixed with the beads and incubated overnight in a rotary wheel (8 rpm) at 4°C. The unbound fraction from the magnetic bead immunoprecipitation were removed and further washed with RIPA buffer and incubated for an additional 2 h in a rotary wheel (8 rpm) at 4°C. A second wash was performed for 5 h on the rotary wheel (18 rpm) at 4°C. Finally, a third washing step was done overnight at 4°C (18 rpm). The bound proteins were eluted by adding 25 μL 1X Laemmli buffer and vortexed vigorously for up to 30 sec. Immunoprecipitated proteins were boiled at 95°C for 5 min and resolved by SDS-PAGE. The ITGA2 corresponding region was excised and digested overnight with either trypsin or chymotrypsin (200 ng) at 37°C. The peptides were extracted and then reconstituted in 0.1% formic acid (20 μL).

#### NanoLC-MS/MSglycoproteomic analysis

All peptide samples were analyzed by reverse-phase high-performance liquid chromatography–electrospray ionization–tandem mass spectrometry (HPLC-ESI-MS/MS) using a Dionex UltiMate 3000 RSLC nano-LC system (Thermo Scientific) connected to an Orbitrap Fusion Tribrid Mass Spectrometer (Thermo Scientific). The mobile phase consisted of 0.1% formic acid (A) and 0.1% formic acid in 80% acetonitrile (B). The autosampler was operated in microliter-pickup injection mode, filling a 20 μl loop with 3 μl of analyte per injection. The sample was loaded onto a reversed-phase trap (Acclaim PepMap 100, 100 μm ID x 2 cm, 5-μm C18 particles), with 0.1% TFA at a flow rate of 6 μL/min. A series of nanoflow gradients (flow rate, 300 nL/min) was used to back-flush the trapped samples onto the nano-LC column (Acclaim PepMap RSLC, 75 μm ID x 15 cm, 2-μm C18 particles) for separation. The peptides were eluted using the following gradient: 2% solvent B in solvent A (from 0 to 6 min), 2%–10% solvent B in solvent A (from 6 to 13 min), 10%–30% solvent B in solvent A (from 13 to 63 min), 30%–50% solvent B in solvent A (from 63 to 70 min), 50%–95% solvent B in solvent A (from 70 to 72 min) and 95% solvent B in solvent A (from 72 to 78 min). The analysis was performed with a total run time of 90 min, including mobile-phase equilibration. Data acquisition was performed in data-dependent mode (DDA), MS1 scan (400-1700 m/z) were performed at a resolution of 60,000 with a 1 × 10^6^ AGC target and maximum injection time of 75 ms. Peptide precursors with charge state 2–7 were sampled for MS2. The instrument was run in top speed mode with a cycle time of 3 s. Dynamic exclusion was enabled with the following settings: repeat count = 1; exclusion duration = 60 s; mass tolerance = ± 10 ppm. Tandem MS was performed by isolation at 2.0 Th with quadrupole isolation. Higher energy collision dissociation (HCD) fragmentation was carried out at 30% normalized collision energy and 10% stepped collision energy. The tandem mass spectra were analyzed with a resolution of 30,000, MS2 AGC target was set to 5 × 10^4^ and the max injection time was 250 ms.

The acquired tandem MS data were processed using Byonic (version 2.16) with the in-build 182 human *N*-glycan library and the 6 common O-glycan libraries. The parameters for enzyme digestion were set to fully specific, with up-to three allowed missed cleavages, and 10 and 20 ppm mass tolerance for precursors and fragment ions, respectively. Carbamidomethylation of cysteine was set as a fixed modification with variable modifications set to include deamidation at Asn and Gln and oxidation of Met. Glycopeptides detected from Byonic interrogation were filtered using the following metrics: Byonic score greater than or equal to 200, a logProb value greater than or equal to 2, and a peptide length greater than at least 4 residues. A maximum of three potential glycosites were allowed for any one glycopeptide. All identified glycopeptides were further manually validated based on the presence of predicted peptide fragments and glycan fragment ions. The precursor ion peak areas of the validated glycopeptides and unoccupied peptides were quantified using Skyline (version 20.1). Quantitation was performed for each glycopeptide for site occupancy and relative abundance by calculating the area under the curve (AUC), with the relative abundance at each site calculated as the area ratio of the peptides bearing a particular glycan over the total peptides of the same peptide sequence.

#### Proteomic analysis of *N*-glycositeITGA2 mutant cells

Protein lysates (400 μg) of KO, KOR, and 10NQ cells were incubated with anti-HA magnetic beads at 4°C overnight on a rotator. Proteins were eluted from anti-HA magnetic beads (CST #11846S) by incubation in 5% SDS, 10 mM TCEP, 100 mM Ammonium Bicarbonate (ABC) for 10 min at 95°C. Eluted proteins were alkylated in 20 mM Iodoacetamide for 30 min and were digested using S-Trap™ micro spin columns (Protifi) according to the manufacturer’s instructions. Phosphoric acid was added (final concentration of phosphoric acid 1.2%) followed by the addition of S-trap buffer (90% methanol, 100 mM TEAB pH 7.1) at a ratio of 6:1. Samples were loaded onto S-trap columns by centrifugation at 4000xg for 1 min followed by three washes with S-trap buffer. Digestion buffer (50 mM TEAB pH 8.0) containing sequencing-grade modified trypsin (1/25, w/w; Promega, Madison, Wisconsin) was added to the S-trap column and incubated for 1 h at 47°C. Peptides were eluted by the consecutive addition and collection for 1 min of 40 μL digestion buffer, 40 μL of 0.2% formic acid and finally 35 μL 50% acetonitrile containing 0.2% formic acid. Samples were dried under vacuum and stored at −20°C until further use.

Dried peptides were resuspended in 0.1% aqueous formic acid and subjected to LC–MS/MS analysis using a LTQ-Orbitrap Elite Mass Spectrometer fitted with an EASY-nLC 1000 (both Thermo Fisher Scientific). Peptides were resolved using a RP-HPLC column (75 μm × 15 cm) packed in-house with C18 resin (ReproSil-Pur C18–AQ, 1.9 μm resin; Dr. Maisch GmbH) at a flow rate of 0.2 μLmin-1. The mass spectrometer was operated in Data-Dependent Acquisition (DDA) mode. The collision energy was set to 35% and one microscan was acquired for each spectrum. The acquired raw-files were imported into the Progenesis QI software (v2.0, Nonlinear Dynamics Limited) to extract peptide precursor ion intensities across all samples. The generated mgf-file was searched using MASCOT against a human database, spiked with the sequence of HA-tagged integrin alpha-2 KOR and 10NQ. Mass tolerance of 10 ppm (precursor) and 0.6 Da (fragments) was set. The database search results were filtered using the ion score to set the false discovery rate (FDR) to 1% on the peptide and protein level, respectively. Quantitative analysis results from label-free quantification were normalized and statically analyzed using the SafeQuant R package v.2.3.4 (https://github.com/eahrne/SafeQuant/) to obtain protein relative abundances (Ahrne et al., 2016). The summarized protein expression values were used for statistical testing of differentially abundant proteins between conditions. Here, empirical Bayes moderated t-tests were applied, as implemented in the R/Bioconductor limma package (http://bioconductor.org/packages/release/bioc/html/limma.html). The resulting p values were adjusted for multiple testing using the Benjamini Hochberg method. All LC-MS analysis runs were acquired from independent biological samples. To meet additional assumptions (normality and homoscedasticity) underlying the use of linear regression models and Student’s t-test MS-intensity signals were transformed from the linear to the log-scale.

### Quantification and statistical analysis

#### Reactome pathway enrichment analysis

Proteomic identified differentially expressed proteins (FDR <1%) with (log2FC > 1 or <−1 and pvalue <0.05) in *N*-glycosites mutant cells were selected for the pathway enrichment analysis. The ReactomePA (Yu and He, 2016) R packages were applied to calculate the Reactome pathway enrichment and visualize the enrichment results as shown in (Figures 5C and 5G).

#### Statistical analysis

All data including error bars are presented as mean ± SD in triplicates unless otherwise stated. Statistical calculations were performed using GraphPad Prism 9.0. Two experimental groups were compared by using unpaired Student’s t-tests. Where more than two groups were compared, a one-way ANOVA with Bonferroni’s correction was used. p values <0.05 were considered statistically significant (∗∗∗, p < 0.001, ∗∗p < 0.01, ∗p < 0.05).

## Data Availability

All data reported in this paper will be shared by the lead contact upon request. This paper does not report on the original code. Any additional information required to reanalyze the data reported herein, is available from the lead contact person upon request.

## Supplemental information

Document S1. Figures S1–S11

Table S1. Analysis of N-glycosylation sites

Table S2. Analysis of O-glycosylation sites

Table S3. Proteomic profiling of ITGA2-edited cells
