# Supplementary material for: Site-specific N-glycosylation of integrin α2 mediates collagen-dependent cell survival
Source: iScience. 2021 Sep 25;24(10):103168. doi: 10.1016/j.isci.2021.103168 (PMC8501769; doi:10.1016/j.isci.2021.103168)
Supplement: Document S1. Figures S1–S11 [file mmc1.pdf]

iScience, Volume *24*

## Supplemental information

# Site-specific *N*-glycosylation of integrin α2 mediates collagen-dependent cell survival

**Yen-Lin Huang, Ching-Yeu Liang, Vera Labitzky, Danilo Ritz, Tiago Oliveira, Cécile Cumin, Manuela Estermann, Tobias Lange, Arun V. Everest-Dass, and Francis Jacob**

Supplementary Figure 1

N-glycosylation

| Position | Potential Scores | N-X-S/T Sequence | Predicted Positivity | Identification |
|---|---|---|---|---|
| 105 | 0.7641 | NVTE | +++ | +/- |
| 112 | 0.5699 | NMSL | + | +/- |
| 343 | 0.6437 | NVSD | ++ | + |
| 432 | 0.4427 | NHSS | - | + |
| 460 | 0.7207 | NYTG | ++ | + |
| 475 | 0.7961 | NITV | +++ | + |
| 699 | 0.7358 | NITL | ++ | + |
| 1057 | 0.6952 | NVTC | ++ | + |
| 1074 | 0.6017 | NVTT | + | + |
| 1081 | 0.4972 | NGTF | - | NA |

Source-version NetNGlyc 1.0 Server
Scores >0.5 are predicted as N-glycosylated and marked positive
NA, no corresponding peptide identify. + positive,
+/- : Identify N-glycan on the same peptide either from site 105 or 112.

O-glycosylation

| Position | Potential Scores | Sequence | Positivity |
|---|---|---|---|
| 99 | 0.7964 | TSTS | + |
| 100 | 0.9179 | S | + |
| 101 | 0.7023 | T | + |
| 102 | 0.7564 | S | + |
| 159 | 0.6367 | SASFS | + |
| 161 | 0.6812 | S | + |
| 1031 | 0.7355 | TSSSVS | + |
| 1032 | 0.7557 | S | + |
| 1033 | 0.5274 | S | + |
| 1034 | 0.7591 | S | + |
| 1036 | 0.8112 | S | + |
| 1039 | 0.7422 | S | + |

source-version NetOGlyc 4.0.0.13
Scores >0.5 are predicted as O-glycosylated and marked positive
No predicted O-glycans are identified

**Supplementary Figure 1. *In silico* prediction of ITGA2 glycosylation sites, related to Figure 2.** The numbers of *N*- and *O*- glycosylation of ITGA2 are predicted using NetNGlyc 1.0 and NetOGlyc 4.0 Server, respectively. Potential scores > 0.5 are predicted as positive glycosylation sites. In total, 10 *N*-glycosylation and 12 *O*-glycosylation sites are predicted (+). Seven *N*-glycosylation sites are confirmed by our mass spectrometry (+).

Supplementary Figure 2

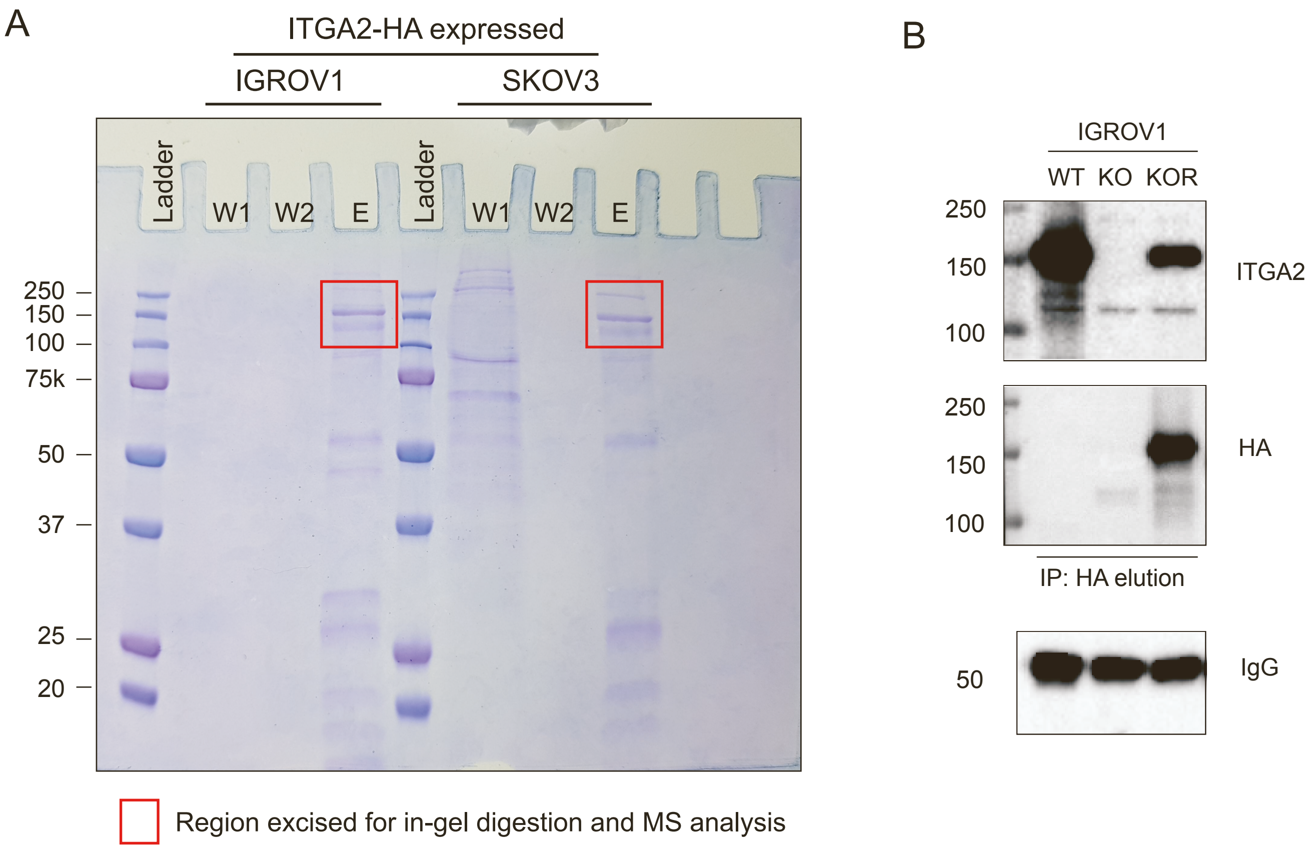


**Supplementary Figure 2. Purification of ITGA2 glycoprotein by immunoprecipitation, related to Figure 2. (A)** 400μg of whole protein lysates of cells expressed with pUltra-Chili-ITGA2-HA were immunoprecipitated with 25μL magnetic anti-HA beads, washed twice with 1XRIPA buffer and eluted with 1X laemmli buffer (W1: 1st wash; W2: 2nd wash, E: 3 μL of eluent). Samples were resolved by SDS-PAGE and stained by coomassie blue. The corresponding region of HA-tagged ITGA2 were cut and subjected to in-gel trypsin digestion before MS analysis. **(B)** Western blot confirmed the purity of ITGA2 glycoprotein after immunoprecipitation.

Supplementary Figure 3

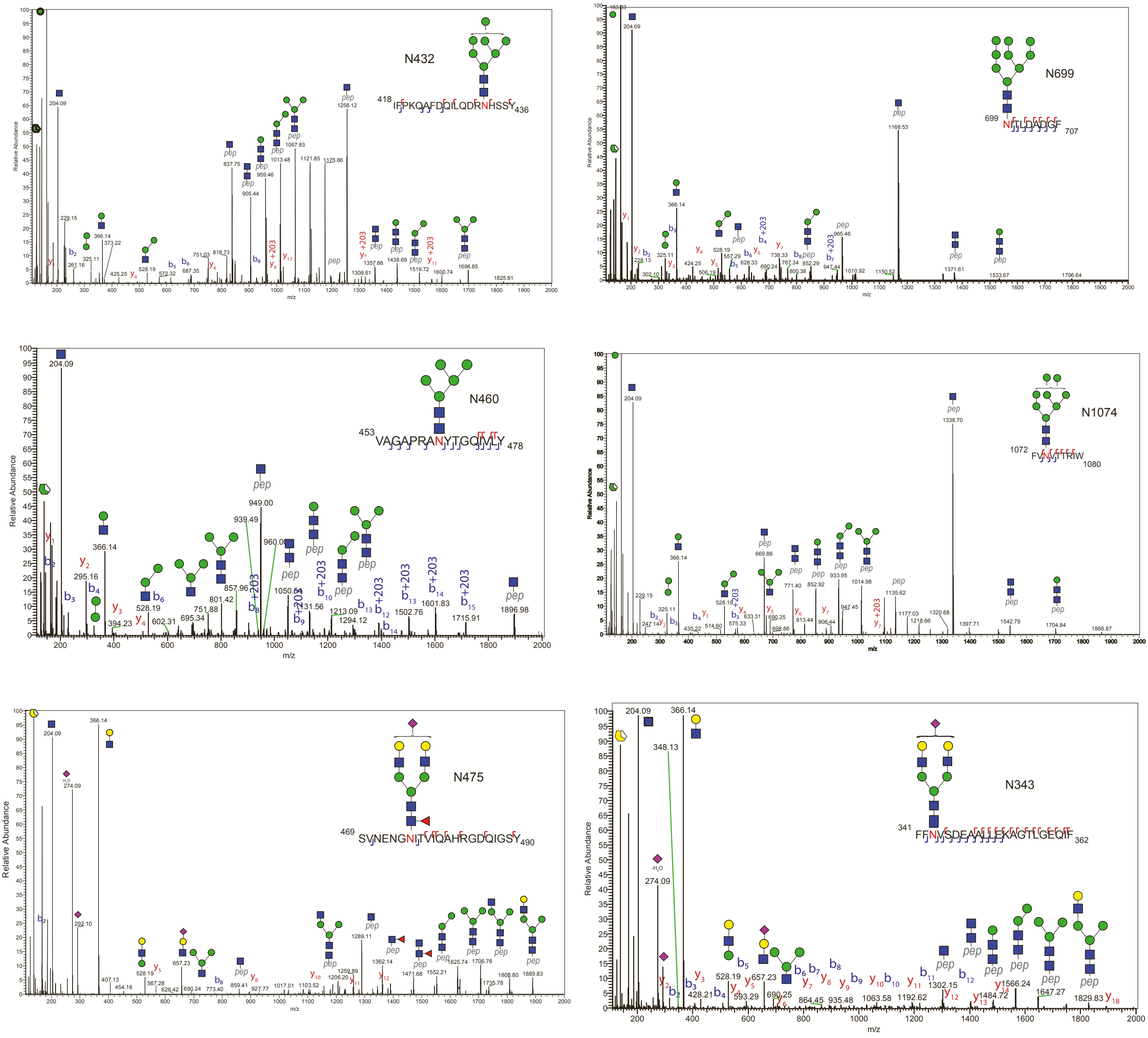


**Supplementary Figure 3. Identification and annotation of ITGA2 *N*-glycosylated peptides, related to Figure 2.** LC-MS/MS-based identification of *N*-glycopeptides is shown corresponding to each *N*-glycosylation site. For each *N*-glycosylation site, the representative step HCD MS2 spectrum is shown to exemplify its identification of glycan composition and peptide sequence. The monosaccharide color code follows the Symbol Nomenclature For Glycans (SNFG).

Supplementary Figure 4

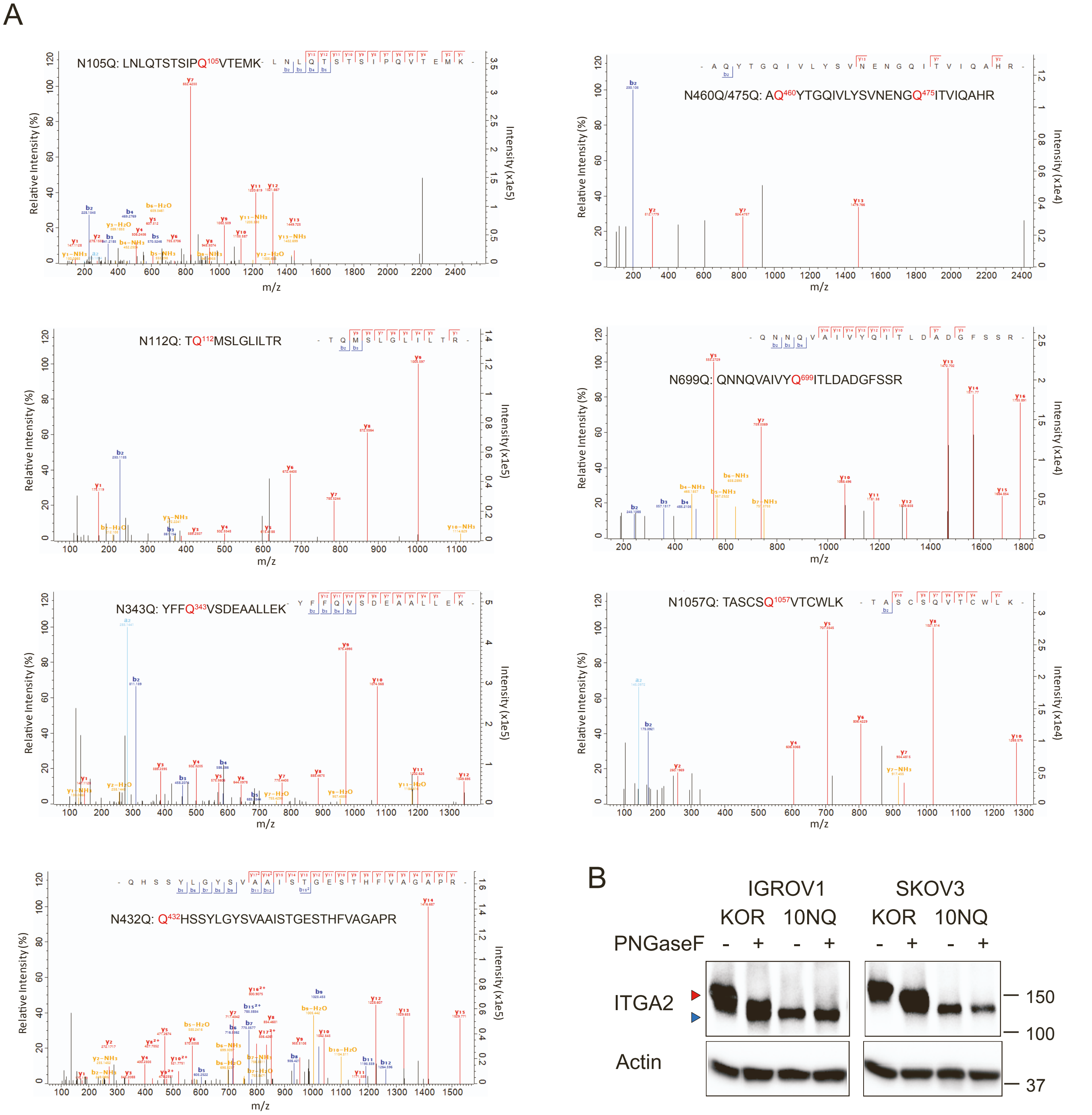


**Supplementary Figure 4. Establishment and confirmation of *N*-glycosite mutant 10NQ cells by mass spectrometry, related to Figure 3. (A)** Representative LC-MS/MS profiles reveal the identification of site-directed mutagenesis of 10NQ in SKOV3 cells (site N1081Q peptide was not detectable). **(B)** Protein lysates of IGROV1 and SKOV3 ITGA2-KOR and non-glycosylated 10NQ cell lines were treated with or without PNGaseF to remove *N*-glycans before subjected to Western blot analysis. Red arrow indicated glycosylated form, Blue arrow indicated deglycosylated ITGA2 with a significant mobility shift.

Supplementary Figure 5

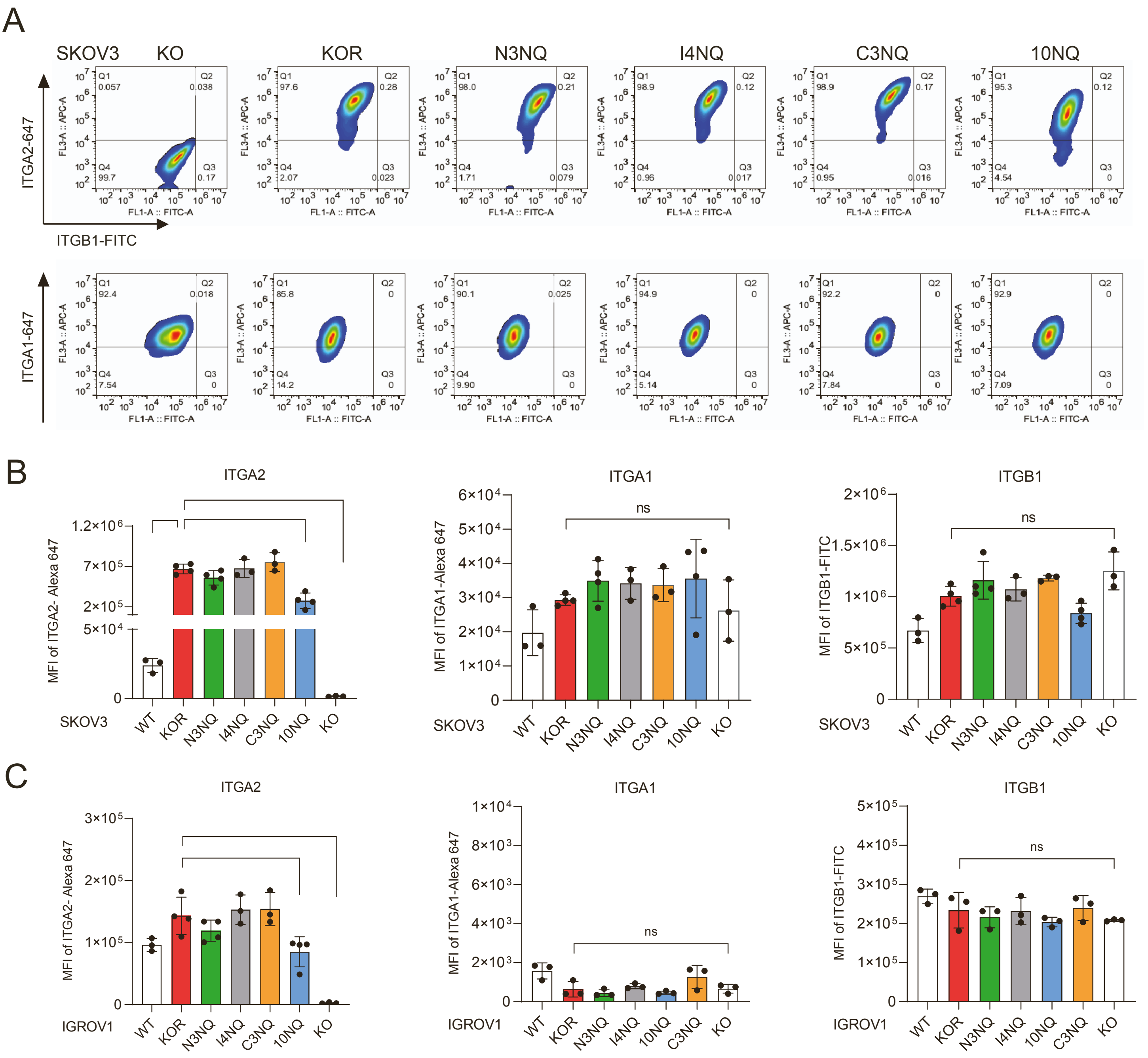


**Supplementary Figure 5. Characterization of the membranous integrin expression in *N*-glycosite mutant cell lines, related to Figure 3. (A)** Representative density plots of cell surface expression of ITGA2, ITGB1 and ITGA1 in WT, KO, KOR and *N*-glycosite mutants (N3NQ, I4NQ, C3NQ, 10NQ) by flow cytometry. **(B)** Bar charts represent mean of ITGA2, ITGA1, and ITGB1 MFI expression (median fluorescence intensity) by flow cytometry. Data are expressed in mean ± SD from three independent experiments (ANOVA * $p < 0.05$, *** $p < 0.001$).

Supplementary Figure 6

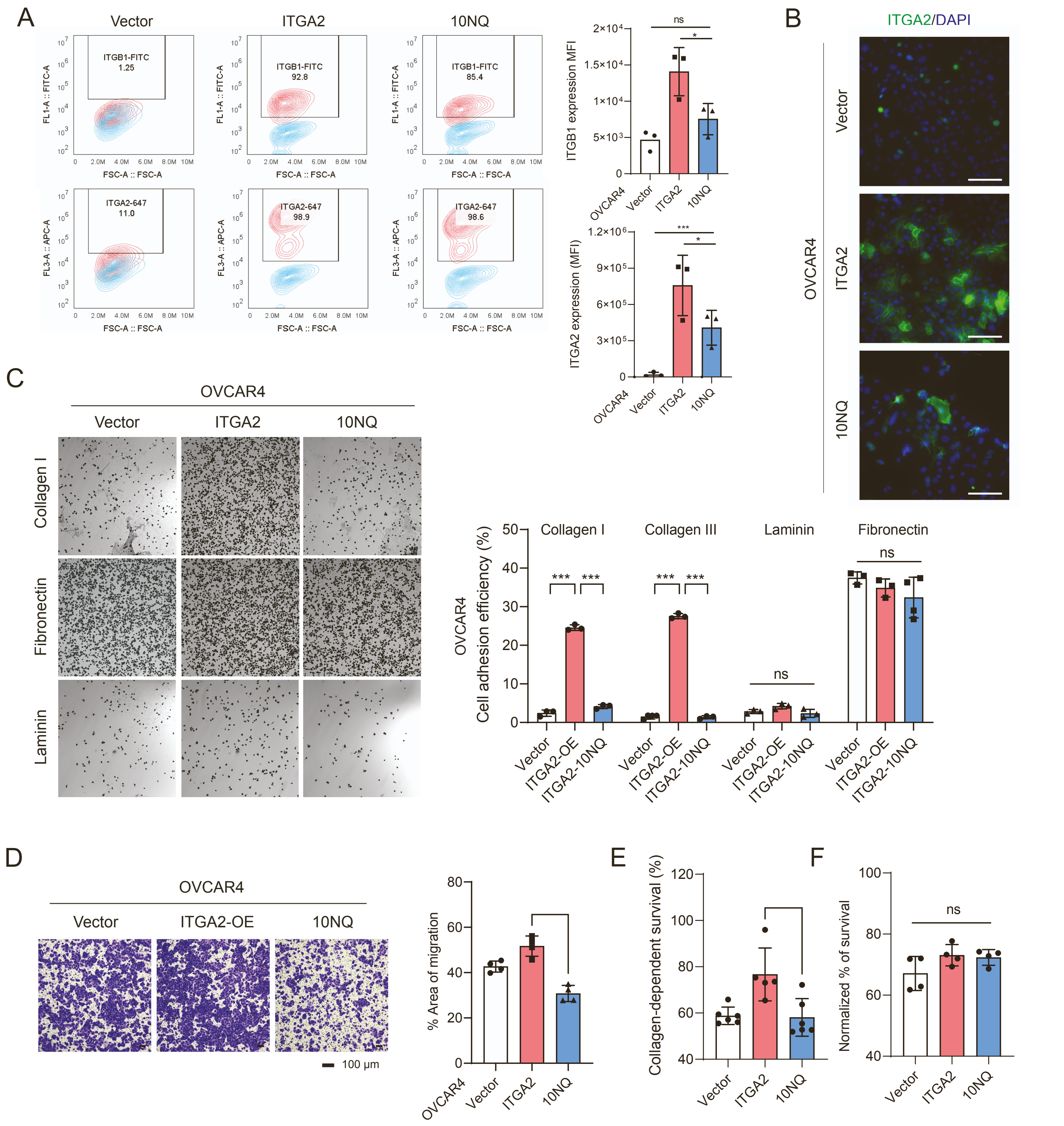


**Supplementary Figure 6. Establishment of ITGA2 overexpression and *N*-glycosite mutant 10NQ OVCAR4 cell lines, related to Figure 3. (A)** Representative density plots of cell surface expression of ITGA2 and ITGB1 in OVCAR4 vector control (pUltra-Chili), ITGA2 overexpressed (pUltra-Chili-ITGA2), and 10NQ *N*-glycosite mutants (pUltra-Chili-10NQ) by flow cytometry. Bar charts represent MFI expression (median fluorescence intensity) by flow cytometry. (ANOVA * $p < 0.05$, *** $p < 0.001$). **(B)** Representative immunofluorescence images of OVCAR4 vector, ITGA2-OE and 10 NQ mutant cells on collagen-coated slide. Cells were stained with anti-ITGA2, and nucleus (DAPI). Scale bar 100 µm. **(C)** Cell-ECM adhesion assay of control, ITGA2-OE and 10NQ-OE OVCAR4 cells. Bar chart shows the percentage of cell adhesion efficiency (ANOVA, *p < 0.05). **(D)** Representative cell migration assay. Cells were fixed and stained after 16 h incubation time. Scale bar 100 µm. Bar charts show the percentage area of migration (ANOVA, ***p < 0.001). **(E)** Collagen-dependent cell survival and **(F)** standard proliferation in plastic culture wells were determined by MTT assay after 48h cultivation in collagen I coated- and plastic plate. Mean ± SD (*p<0.05).

Supplementary Figure 7

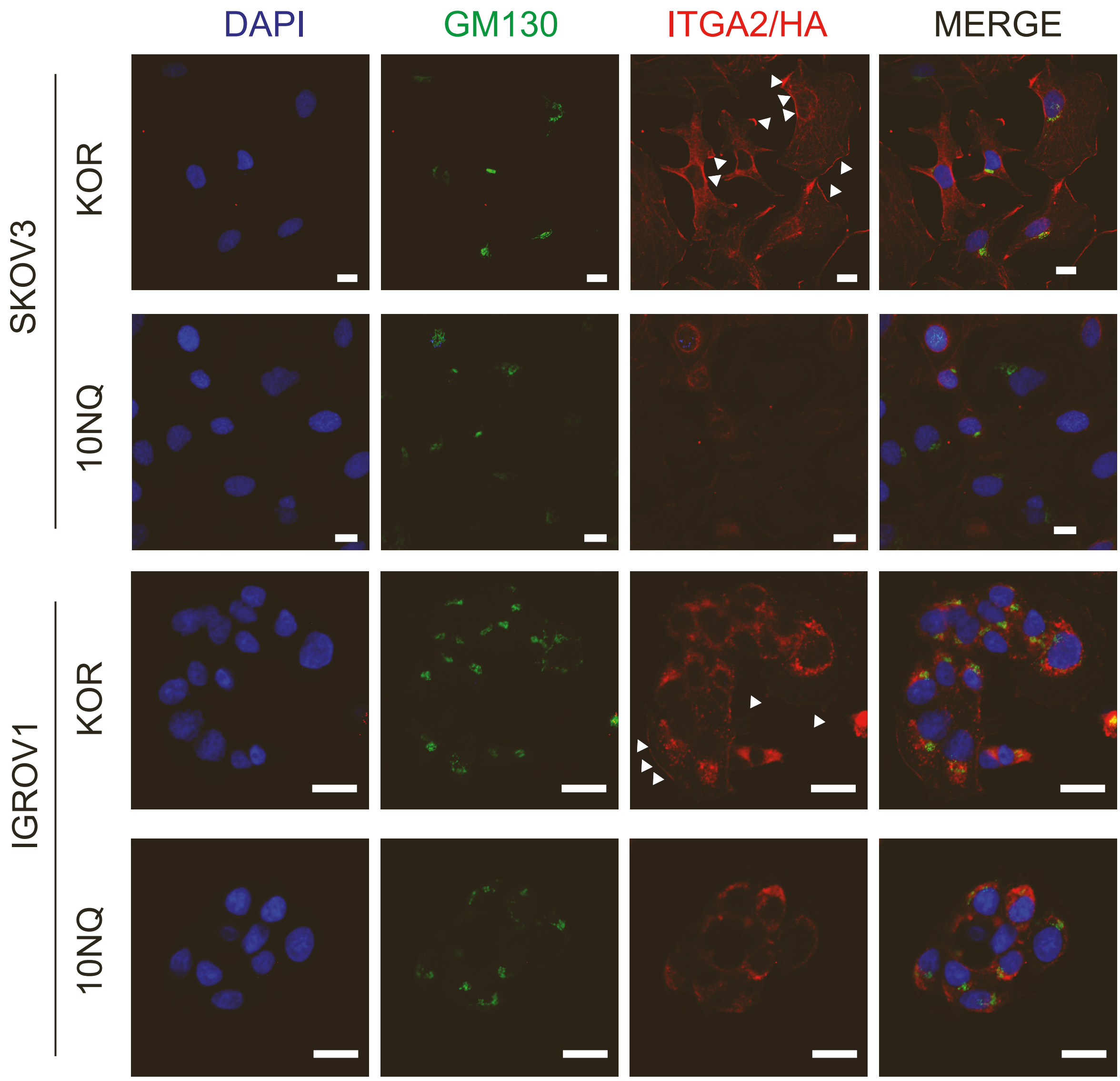


**Supplementary Figure 7. Loss of site-specific *N*-glycosylation affects membranous ITGA2 expression, related to Figure 3.** Representative immunofluorescence images of ITGA2-KOR and 10 NQ mutant cells on collagen-coated slide. Both IGROV1 and SKOV3 mutant cells were co-stained with anti-HA (ITGA2-HA, red), anti-GM130 (cis-Golgi marker, green) and nucleus (DAPI). Scale bar 20 μm. White arrowheads show the membrane protrusions.

Supplementary Figure 8

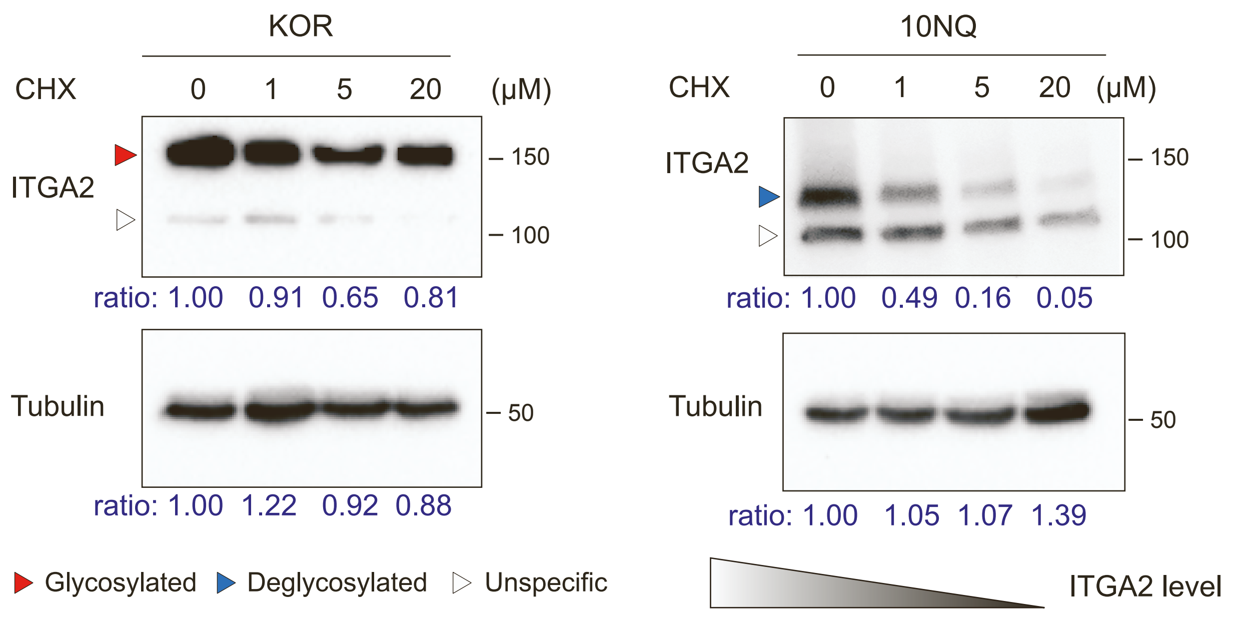


**Supplementary Figure 8. Cycloheximide induces ITGA2 protein turnover in a dose-dependent manner, related to Figure 4.** IGROV1 KOR and 10NQ cells were treated with protein synthesis inhibitor cycloheximide (CHX) at indicated dose for 16h before harvested protein lysates and subjected to Western blot analysis. A significant reduction of nonglycosylated 10NQ ITGA2 expression was identified in a dose-dependent manner. The protein expression ratio of each sample is determined by densitometry using Image Lab 6.01 Software.

Supplementary Figure 9

A

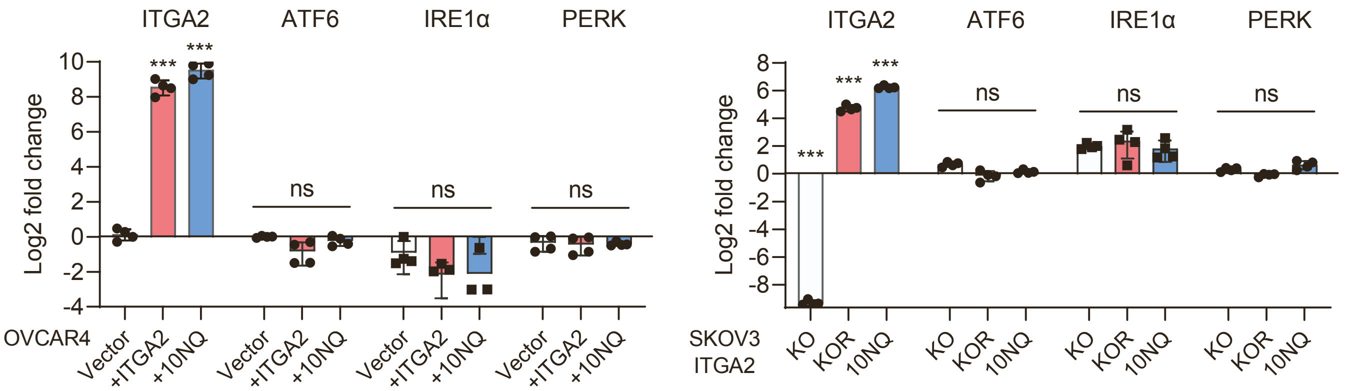


B

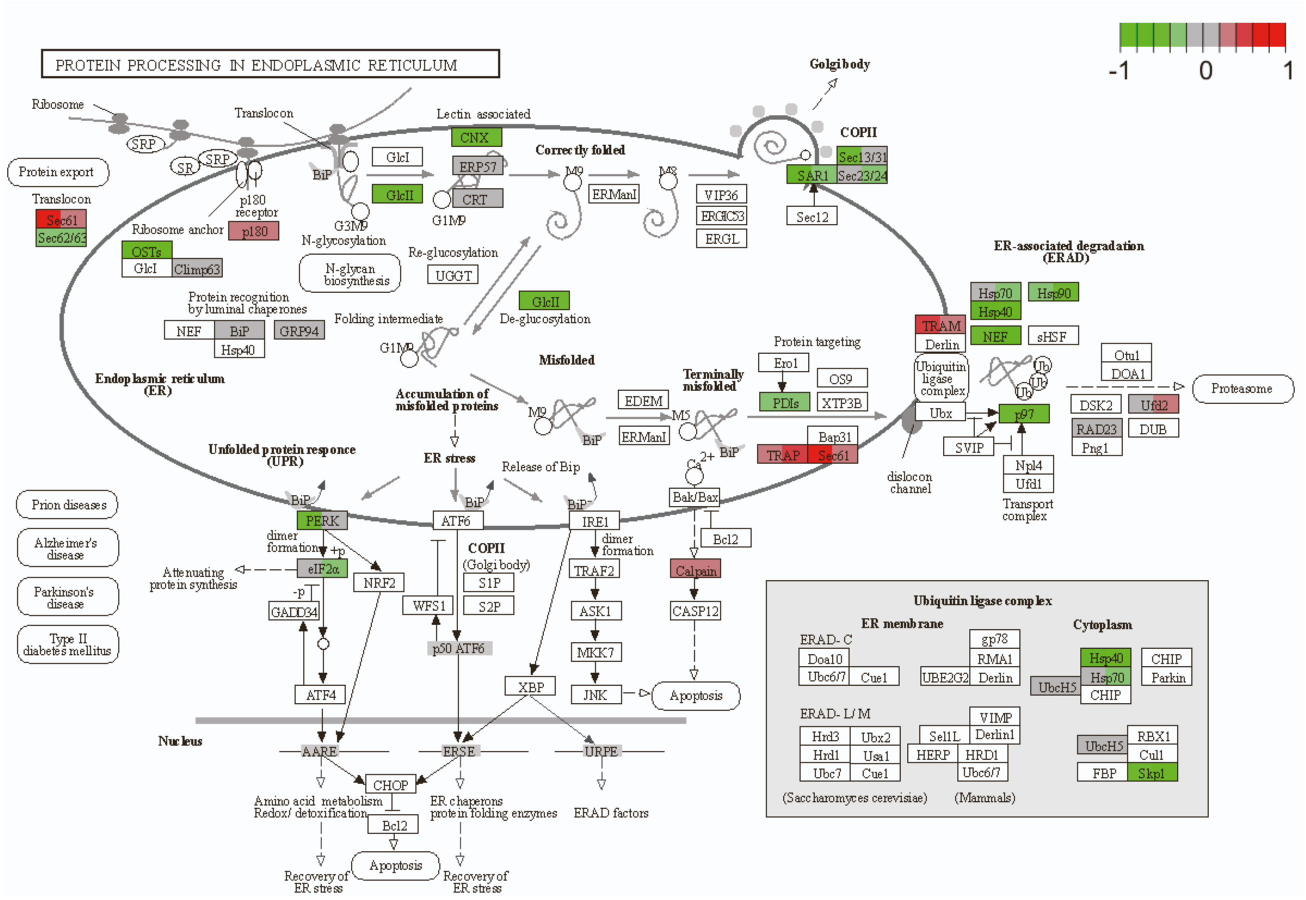


**Supplementary Figure 9. Overexpression of ITGA2 and glycosite-mutant ITGA2 has marginal impact on ER/UPR stress signaling, related to Figure 4. (A)** Quantitative PCR analysis of ER/UPR stress related gene in SKOV3 KO, KOR and 10NQ cells as well as OVCAR4 vector (pUltra-Chili), ITGA2 overexpressed (pUltra-Chili+ITGA2), and 10NQ overexpressed cells (pUltra-Chili+10NQ). The Log2 fold change of gene expression were normalized to IGROV1 WT cells. (ANOVA * $p < 0.05$). **(B)** KEGG PathView depicts up- (red) and down-regulated (green) proteins of the hsa04141:protein processing in endoplasmic reticulum pathway comparing non-glycosylated 10NQ *versus* glycosylated ITGA2 KOR cells. Pair-wise gene expression changes are transformed and expressed by log2 ratio.

Supplementary Figure 10

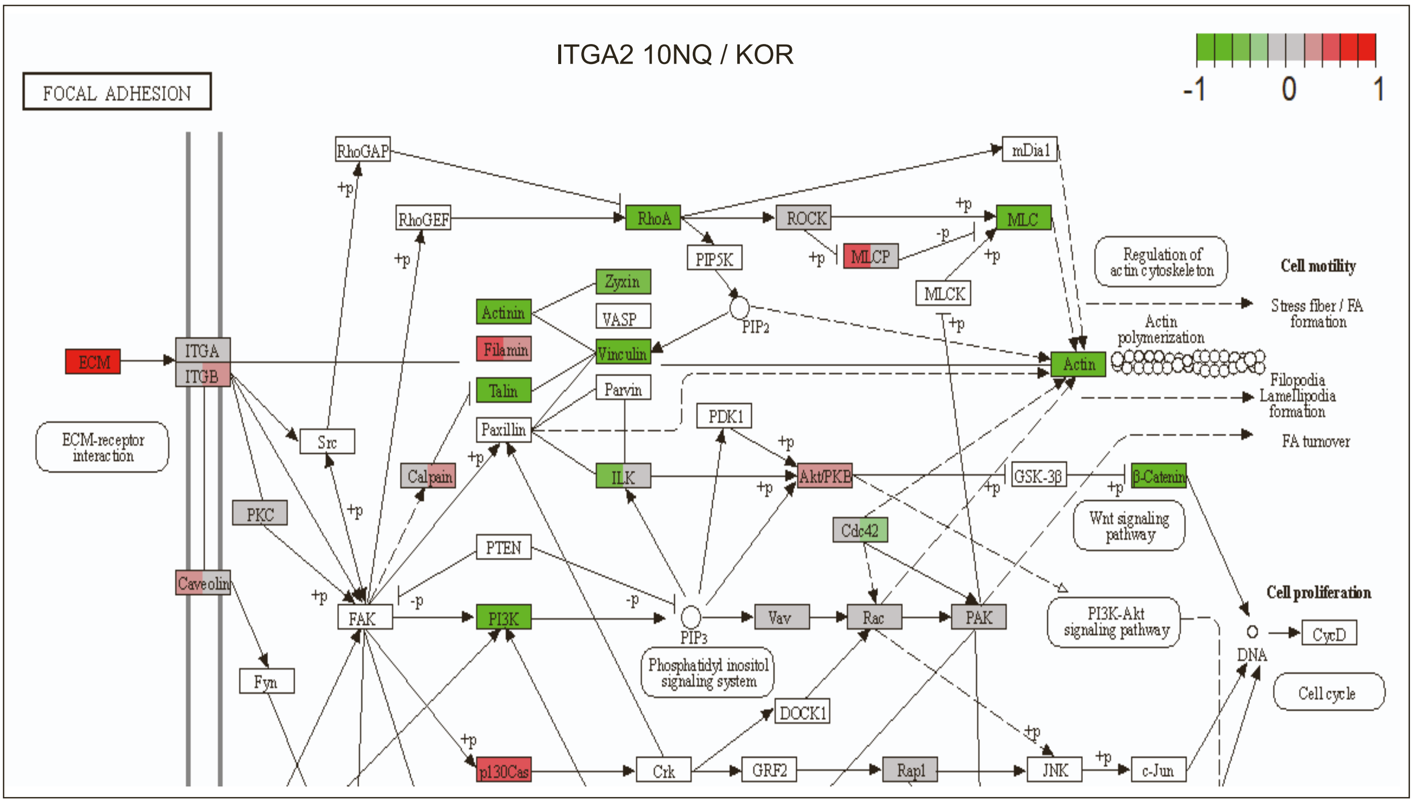


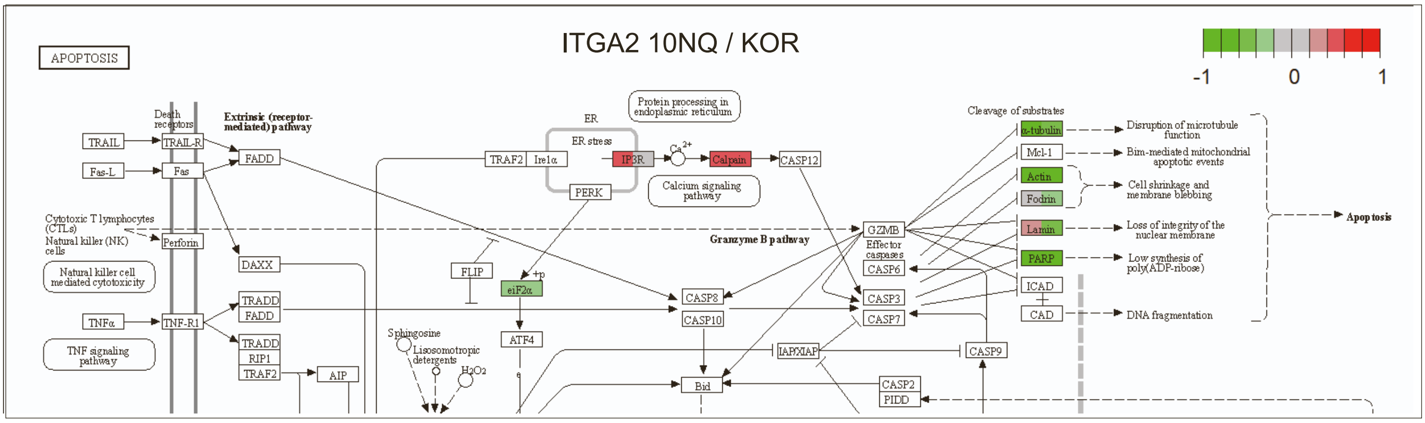


**Supplementary Figure 10. ITGA2 glycosylation-dependent KEGG signaling, related to Figure 5.** KEGG PathView depicts up- (red) and down-regulated (green) proteins of the hsa04510:focal adhesion pathway and hsa04210 apoptosis pathway comparing non-glycosylated 10NQ *versus* glycosylated ITGA2 KOR cells. Pair-wise gene expression changes are transformed and expressed by log2 ratio.

Supplementary Figure 11

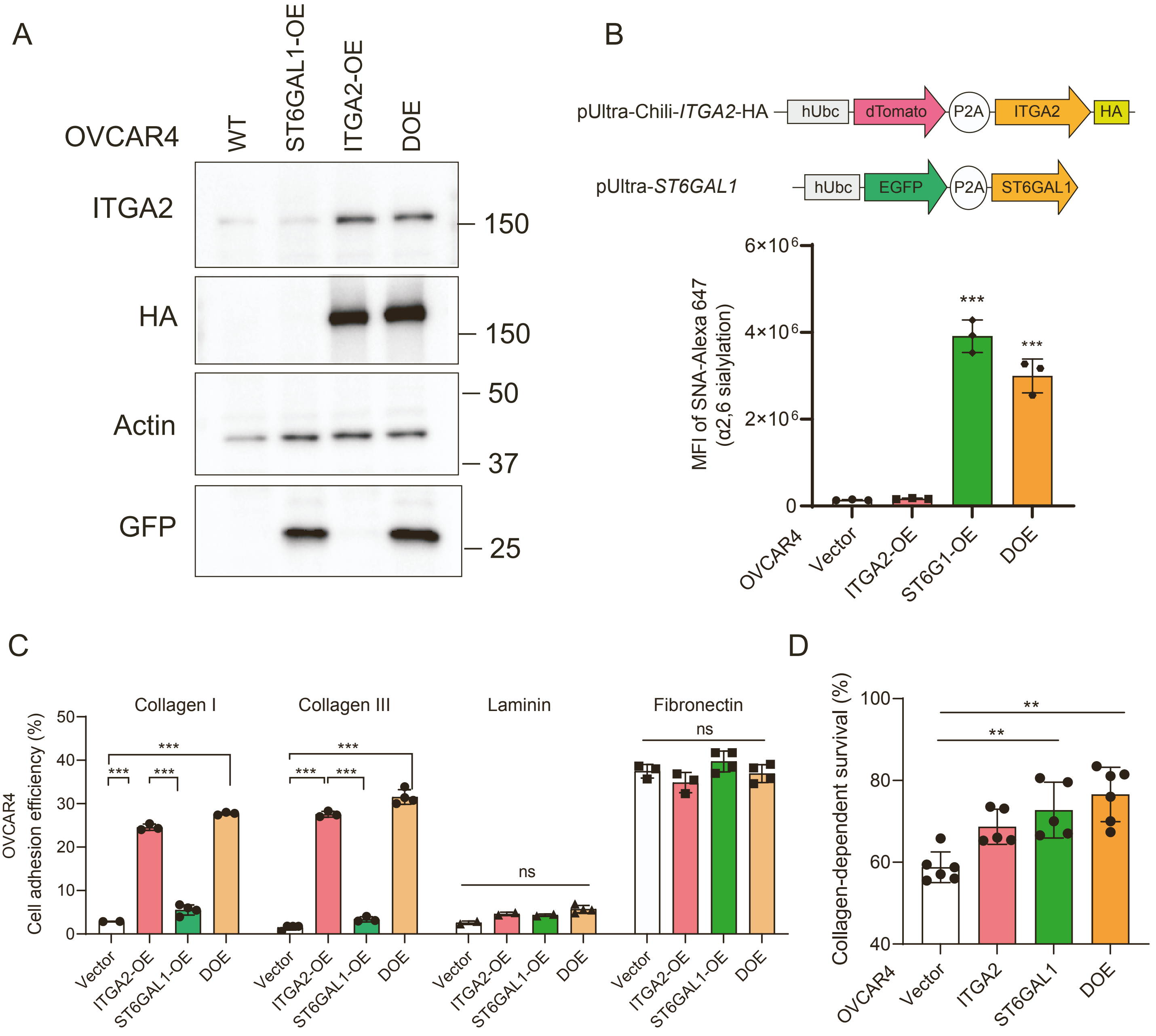


**Supplementary Figure 11. Induce expression of ST6GAL1 and ITGA2 promotes cancer cell adhesion to collagen and survival in OVCAR4, related to Figure 6. (A)** Western blot analysis confirmed the overexpression (OE) of ITGA2, ST6GAL1, and DOE (double overexpression) in OVCAR4 cell line. **(B) (C)** Cell surface sialylation was measured by flow cytometry as previously described. (ANOVA ***$p$ <0.001). **(C)** Cell adhesion efficiency represented by mean±SD (ANOVA, ***$p$ <0.001). **(D)** Collagen-dependent survival were determined by MTT assay after 48h cultivation in collagen I-coated plate. Mean ± SD (**p<0.01).
